# Co-assembly of Viral Envelope Glycoproteins Regulates Their Polarized Sorting in Neurons

**DOI:** 10.1371/journal.ppat.1004107

**Published:** 2014-05-15

**Authors:** Rafael Mattera, Ginny G. Farías, Gonzalo A. Mardones, Juan S. Bonifacino

**Affiliations:** Cell Biology and Metabolism Program, Eunice Kennedy Shriver National Institute of Child Health and Human Development, National Institutes of Health, Bethesda, Maryland, United States of America; Icahn School of Medicine at Mount Sinai, United States of America

## Abstract

Newly synthesized envelope glycoproteins of neuroinvasive viruses can be sorted in a polarized manner to the somatodendritic and/or axonal domains of neurons. Although critical for transneuronal spread of viruses, the molecular determinants and interregulation of this process are largely unknown. We studied the polarized sorting of the attachment (NiV-G) and fusion (NiV-F) glycoproteins of Nipah virus (NiV), a paramyxovirus that causes fatal human encephalitis, in rat hippocampal neurons. When expressed individually, NiV-G exhibited a non-polarized distribution, whereas NiV-F was specifically sorted to the somatodendritic domain. Polarized sorting of NiV-F was dependent on interaction of tyrosine-based signals in its cytosolic tail with the clathrin adaptor complex AP-1. Co-expression of NiV-G with NiV-F abolished somatodendritic sorting of NiV-F due to incorporation of NiV-G•NiV-F complexes into axonal transport carriers. We propose that faster biosynthetic transport of unassembled NiV-F allows for its proteolytic activation in the somatodendritic domain prior to association with NiV-G and axonal delivery of NiV-G•NiV-F complexes. Our study reveals how interactions of viral glycoproteins with the host's transport machinery and between themselves regulate their polarized sorting in neurons.

## Author Summary

Neurons are highly polarized cells exhibiting somatodendritic and axonal domains with distinct protein and lipid compositions. Some enveloped viruses target neurons by binding of the viral envelope glycoproteins to neuronal surface receptors. The ensuing fusion of the viral and neuronal membranes delivers the genetic material of the virus into the neurons. During viral replication in neurons, newly synthesized envelope glycoproteins are sorted to the somatodendritic and/or axonal domains. Although critical for viral propagation, the mechanisms responsible for this sorting are largely unknown. We studied the neuronal sorting of the attachment (NiV-G) and fusion (NiV-F) glycoproteins of Nipah virus, a pathogen that causes fatal human encephalitis. When analyzed individually, NiV-G was delivered to both the axonal and somatodendritic domains. In contrast, NiV-F was exclusively targeted to the somatodendritic domain by virtue of interaction of specific signals in this protein with AP-1, a component of the neuronal protein transport machinery. Assembly with NiV-G, however, abolished somatodendritic sorting of NiV-F due to incorporation of complexes into axon-bound vesicles. Thus, coordinated interactions of viral glycoproteins with the host's sorting machinery and between themselves allow temporal and spatial regulation of their distribution in neurons. We propose that this coordination facilitates viral spread among neurons.

## Introduction

Neurons are polarized cells comprising somatodendritic and axonal domains with unique structural and functional properties (reviewed in [1]–[3]). Sorting of specific assortments of membrane proteins and lipids to these domains is essential for neuronal function. Members of many virus families have developed strategies to invade the nervous system and cause acute or persistent neurovirulence [4], [5]. Upon infection or transfection, transmembrane proteins encoded by neuroinvasive viruses also undergo polarized sorting in neurons [6]–[8]. The polarized distribution of viral proteins in neurons is critical for the life cycle and transneuronal spread of viruses [5], [8], [9], and must be accurately coordinated with biosynthetic processing in organelles located in the soma. The localization of viral glycoproteins and matrix proteins, in particular, can direct polarized release of viruses from epithelial cells [10]–[15]. The axonal or somatodendritic distribution of viral membrane proteins must result from differences in their interaction with repurposed components of the neuronal sorting machinery. These primary interactions could also be regulated by other proteins encoded in the viral genome. The analysis of these two layers of regulation is key to understanding the sorting of viral proteins in neurons.

**Figure 1:**
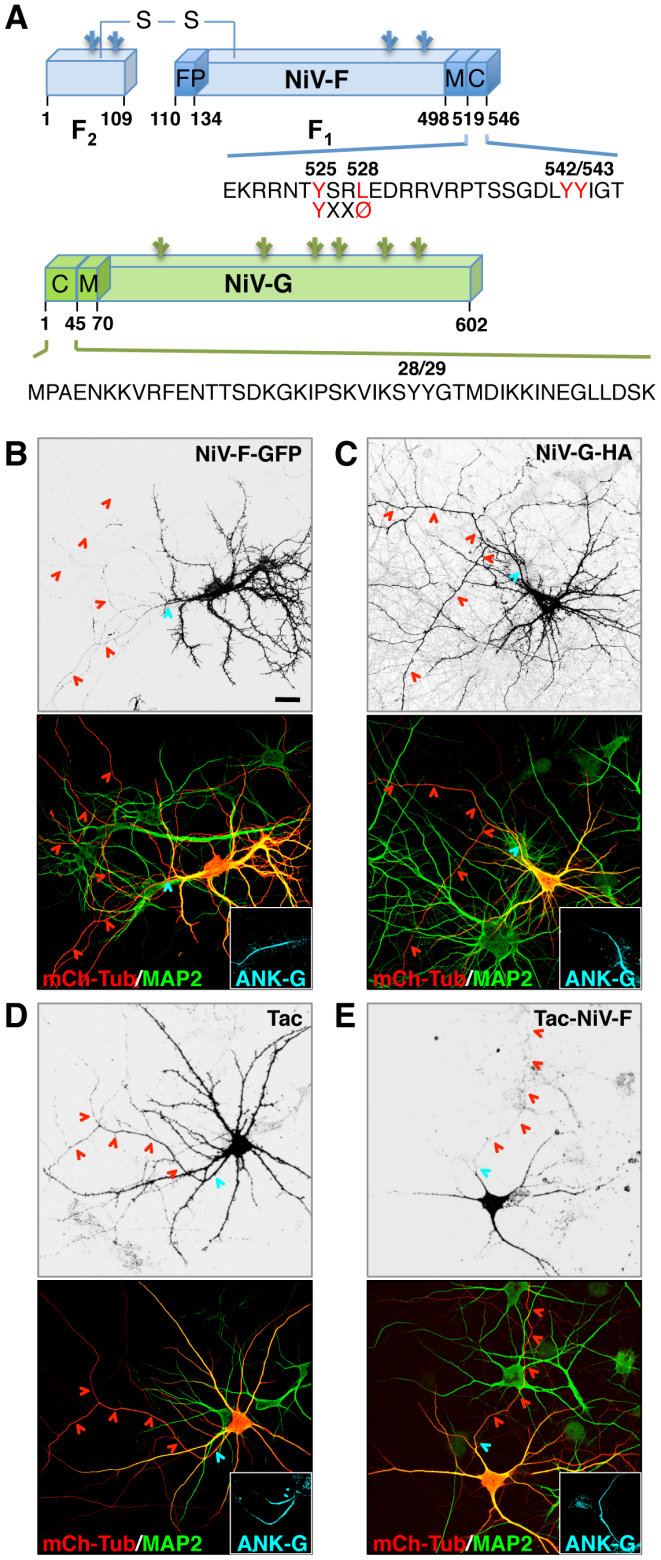
Somatodendritic sorting of NiV-F mediated by its cytosolic tail. (A) Schematic representation of NiV-F and NiV-G indicating the NIV-F fusion peptide (FP), transmembrane (M) and cytosolic (C) domains, and amino-acid sequences of the cytosolic domains. The F_2_-F_1_ active form of NiV-F generated by cathepsin L- or B-mediated cleavage of the F_0_ inactive precursor is stabilized by a disulfide (S-S) bridge [29], [30]. Utilized N-linked glycosylation sites [26]–[28] are indicated by branched lines. The YXXØ motif (YSRL, residues 525–528) and a YY pair (residues 542 and 543) in the NiV-F tail are highlighted in red. (B–E) Rat hippocampal neurons were co-transfected on DIV4 with plasmids encoding NiV-F-GFP, NiV-G-HA, Tac or Tac-NiV-F, and mCherry-tubulin (mCh-Tub, marker of both dendrites and axons), fixed on DIV10, and immunostained with rabbit anti-MAP2 and goat anti-ankyrin-G (ANK-G) (to identify dendrites and the AIS, respectively) and with mouse anti-HA or mouse anti-Tac antibodies (to visualize NiV-G-HA or Tac-based constructs). Cells were imaged by confocal microscopy. Grayscale images correspond to NiV-F-GFP fluorescence (B), anti-HA (C) or anti-Tac (D, E) staining. Merged color pictures at the bottom of all panels display mCh-Tub fluorescence (red) and anti-MAP2 (green) staining (axons appear red due to mCh-Tub labeling, while dendrites are yellow due to co-labeling by mCh-Tub and MAP2). Insets show anti-ANK-G labeling (AIS shown in cyan). The AIS and axons are marked by cyan and red arrowheads, respectively. Scale bar: 20 µm. Quantitative analysis of NiV-F and NiV-G polarized sorting was performed through calculation of the dendrite/axon (D/A) polarity index (Table 1).

The Nipah virus (NiV) is a recently identified, highly pathogenic, paramyxovirus (*Henipavirus* genus) that exhibits broad host and cell tropism and infects various human cell types [16], [17]. NiV enters the body via the upper respiratory tract; whereas epithelial cells are important during the initial phase of the infection, vascular endothelial cells are critical during the systemic phase that results in widespread vasculitis and viremia [18], [19]. Damage to the vasculature allows NiV to cross the blood-brain barrier and infect neurons, and less frequently glia, causing encephalitis with high (∼75%) mortality rate in humans [18]. The two glycoproteins in the viral envelope, NiV fusion (NiV-F) and NiV attachment or receptor-binding (NiV-G), are key to invasion of host cells (reviewed in [20]–[22]). High-affinity binding of NiV-G to ephrin-B2 or -B3 represents the first step in the recognition of host cells by the virus [23]–[25]. This binding is associated with “triggering” of NiV-F, resulting in fusion of the viral envelope with the host cell membrane [22]. NiV-F and NiV-G mRNAs are translated at ER-associated ribosomes, co-translationally inserted into the ER where they undergo N-linked glycosylation and subsequently transported to the plasma membrane [20], [26]–[28]. NiV-F is synthesized as a fusion-inactive precursor (NiV-F_0_), which undergoes endocytosis followed by cathepsin L- or B-dependent cleavage to the active NiV F_2_-F_1_ form in acidic endosomes before recycling to the plasma membrane (Figure 1A; [20], [29], [30]). Several studies have addressed the polarized sorting of NiV proteins in epithelial and endothelial cells and their role in cell-to-cell fusion, syncytia formation and virus spread [15], [31], [32]. However, despite the fact that encephalitis is a hallmark of NiV infection in humans, to date no studies have addressed the neuronal sorting of proteins encoded by this virus.

We have studied the sorting of NiV-F and NiV-G glycoproteins in rat hippocampal neurons. We observed that NiV-F localizes to the somatodendritic domain in a manner dependent on the interaction of tyrosine-based signals in its cytosolic tail with the clathrin adaptor complex AP-1. NiV-G, in contrast, exhibits a non-polarized distribution to both axons and dendrites and does not interact with AP-1. Importantly, co-expression with NiV-G causes NiV-F to lose its somatodendritic polarity, becoming evenly distributed between the somatodendritic and axonal domains. This redistribution is due to the incorporation of NiV-F into axonal transport carriers in the presence of NiV-G. We propose that faster biosynthetic transport of NiV-F allows this protein to be singly transported in an AP-1-dependent manner to the somatodendritic domain, where cathepsin L and B reside. The proteolytically activated NiV-F can then interact with the more slowly transported NiV-G, to form a complex that is delivered to the axonal domain. Thus, coordinated interactions of NiV-F with the host's polarized sorting machinery and with NiV-G allows temporal and spatial control of the transport of active NiV-F•NiV-G complexes to the axonal domain. Our study reveals how interactions of viral envelope glycoproteins between themselves and with the neuronal transport machinery regulate their polarized sorting.

## Results

### Somatodendritic sorting of NiV-F is mediated by tyrosine-based signals in the cytosolic tail

**Figure 2:**
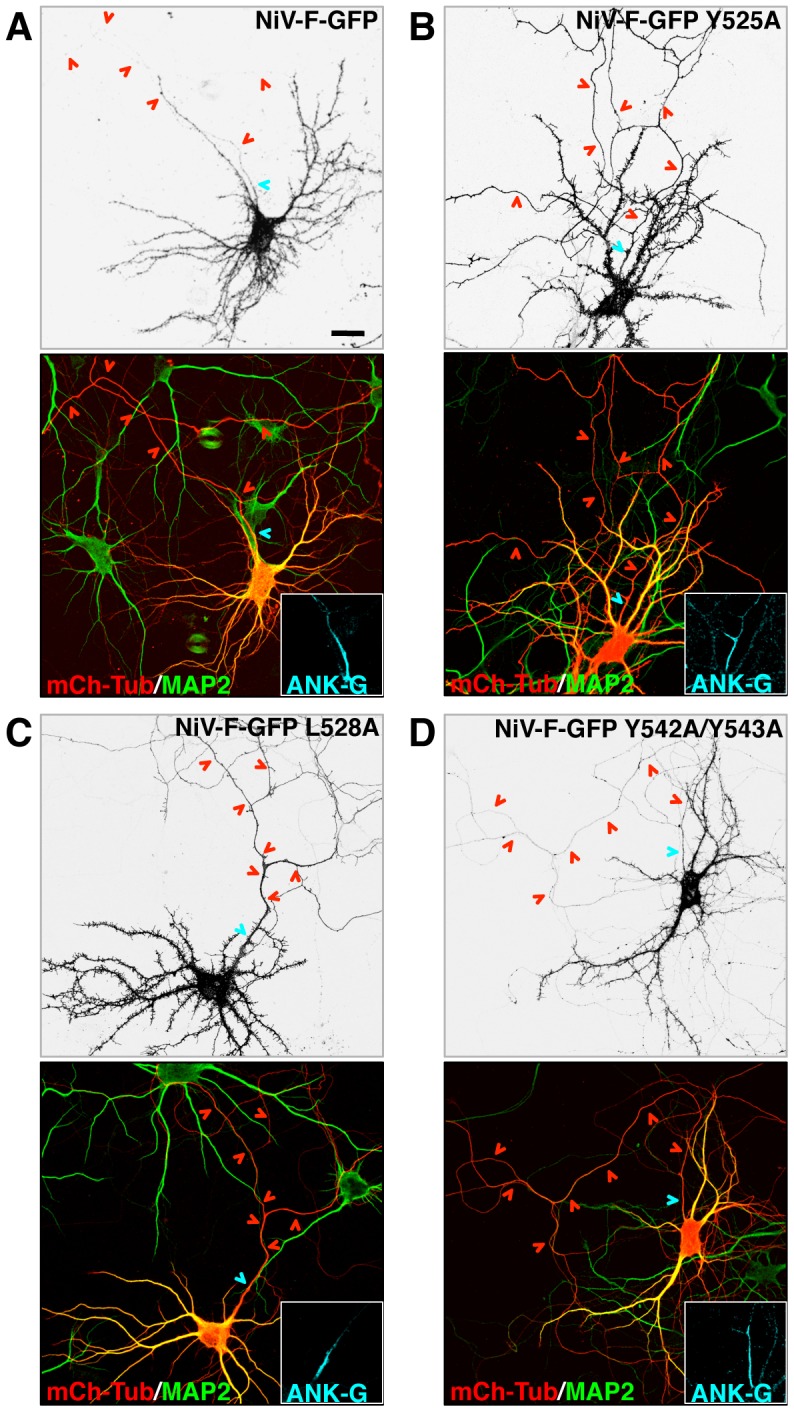
Involvement of a YXXØ motif and a YY pair in somatodendritic sorting of NiV-F. (A–D) Rat hippocampal neurons were co-transfected with the indicated NiV-F-GFP constructs and mCh-Tub and immunostained with rabbit anti-MAP2 and goat anti-ANK-G. Grayscale images depict the fluorescence of the indicated NiV-F-GFP constructs; labeling in color images is as indicated in the legend to Figure 1. Cyan and red arrowheads show the position of AIS and axons, respectively. Scale bar: 20 µm. D/A polarity indexes for all constructs are summarized in Table 1.

To analyze the distribution of the Nipah virus glycoproteins NiV-F and NiV-G (Figure 1A) in neurons, primary cultures of embryonic rat hippocampal neurons were transfected at day-in-vitro 4 (DIV4) with plasmids encoding NiV-F-GFP or NiV-G-HA and examined at day-in-vitro 10 (DIV10) by confocal microscopy. The entire neuronal cytoplasm (soma, dendrites and axon) was visualized by co-expression of mCherry-tubulin (mCh-Tub) and specific neuronal domains were identified by immunostaining with antibodies to MAP2 (dendrites) and Ankyrin-G (ANK-G) (axon initial segment, AIS). We observed that NiV-F-GFP localized to the soma and dendrites (i.e., the somatodendritic domain) but was excluded from the axon (Figure 1B) (dendrite to axon polarity index (D/A): 8.3±2.0; Table 1). In contrast, NiV-G-HA localized to both the somatodendritic and axonal domains (Figure 1C) (D/A: 1.0±0.2; Table 1). Since sorting of transmembrane proteins in the endomembrane system is often dependent on signals in their cytosolic tails [33], [34], we tested whether the NiV-F cytosolic tail contained information for somotodendritic sorting. To this end, we compared the intracellular distribution of the interleukin-2 receptor α subunit (referred to as Tac) and that of a chimeric protein having the extracellular and transmembrane domains of Tac and the NiV-F cytosolic tail (Tac-NiV-F). We found that whereas full-length Tac localized to both the somatodendritic and axonal domains (Figure 1D) (D/A: 1.1±0.2; Table 1), Tac-NiV-F was restricted to the somatodendritic domain (Figure 1E) (D/A: 6.7±1.8; Table 1). The NiV-F cytosolic tail contains a YXXØ motif (X is any amino acid and Ø a bulky hydrophobic amino acid) (YSRL; residues 525–528) (Figure 1A) characteristic of signals that mediate various intracellular sorting events [33], [34], including somatodendritic sorting [35], [36]. Mutation of Y525 or L528 in this motif to alanine resulted in non-polarized distribution of NiV-F-GFP (Figure 2 A–C) (D/A: 0.9±0.2 and 1.1±0.2, respectively; Table 1). The NiV-F cytosolic tail has two other tyrosine residues, the contiguous Y542 and Y543, which are not part of YXXØ motifs (Figure 1A). Individual substitution of these tyrosine residues by alanine did not affect somatodendritic sorting of NiV-F-GFP (data not shown), but their combined substitution resulted in partial loss of NiV-F polarity (Figure 2D) (D/A: 4.4±1.2; Table 1). These analyses thus demonstrated that NiV-F, but not NiV-G, is sorted to the somatodendritic domain of hippocampal neurons mainly by virtue of tyrosine-based signals, including a YXXØ motif, in its cytosolic tail.

**Table 1:** Quantification of NiV-F and NiV-G sorting into somatodendritic and axonal domains of rat hippocampal neurons.

	D/A Polarity Index
NiV-F-GFP	8.3±2.0 (30)
NiV-G-HA	1.0±0.2 (30)*
Tac	1.1±0.2 (25)
Tac-NiV-F	6.7±1.8 (27)§
NiV-F-GFP Y525A	0.9±0.2 (27)*
NiV-F-GFP L528A	1.1±0.2 (30)*
NiV-F-GFP Y542A/Y543A	4.4±1.2 (32)*
NiV-F-GFP+μ1A-HA WT	7.9±2.0 (30)
NiV-F-GFP+μ1A-HA A-site mutant	1.6±0.4 (30)*
NiV-F-GFP+μ2-HA WT	8.4±1.8 (27)
NiV-F-GFP+μ2-HA A-site mutant	5.3±3.1 (39)*
NiV-F-GFP+μ3-HA WT	7.8±1.9 (25)
NiV-F-GFP+μ3-HA A-site mutant	8.2±1.9 (25)
NiV-F-GFP+μ4-HA WT	8.1±2.0 (25)
NiV-F-GFP+μ4-HA A-site mutant	7.8±2.0 (27)
NiV-F-GFP+μ4-HA B-site mutant	8.5±1.8 (25)
NiV-F-GFP (+NiV-G-HA)	1.3±0.2 (20)*
NiV-G-HA (+NiV-F-GFP)	1.2±0.4 (20)*
NiV-F-GFP-Δ104–109	7.5±2.0 (25)
NiV-F-GFP-Δ104–109 (+NiV-G-HA)	1.2±0.3 (22)‡
NiV-F-GFP (+NiV-G-mCh)+μ2-HA WT	1.4±0.3 (22)*
NiV-F-GFP (+NiV-G-mCh)+μ2-HA A-site mutant	1.5±0.4 (27)*

Dendrite to axon (D/A) polarity indexes were calculated as described in Materials and Methods. Values are expressed as mean **±** SD (*n*) (*n*; number of cells analyzed).

The notation NiV-F-GFP (+NiV-G-HA) refers to the NiV-F-GFP polarity index in cells co-expressing NiV-G-HA. The same applies to the NiV-G-HA (+NiV-F-GFP) notation.

Statistical significance for all groups including NiV-F-GFP and NiV-G-HA constructs was calculated by one-way ANOVA followed by Dunnett's test.

(*)*P*<0.01 when compared to NiV-F-GFP. Significance between group pairs including Tac or NiV-F-GFP Δ104–109 constructs was calculated by Student's *t*-test.

(§) *P*<0.01 when compared to Tac;

(‡) *P*<0.01 when compared to NiV-F-GFP Δ104–109.

### Interaction of the NiV-F cytosolic tail with μ subunits of adaptor protein (AP) complexes

**Figure 3:**
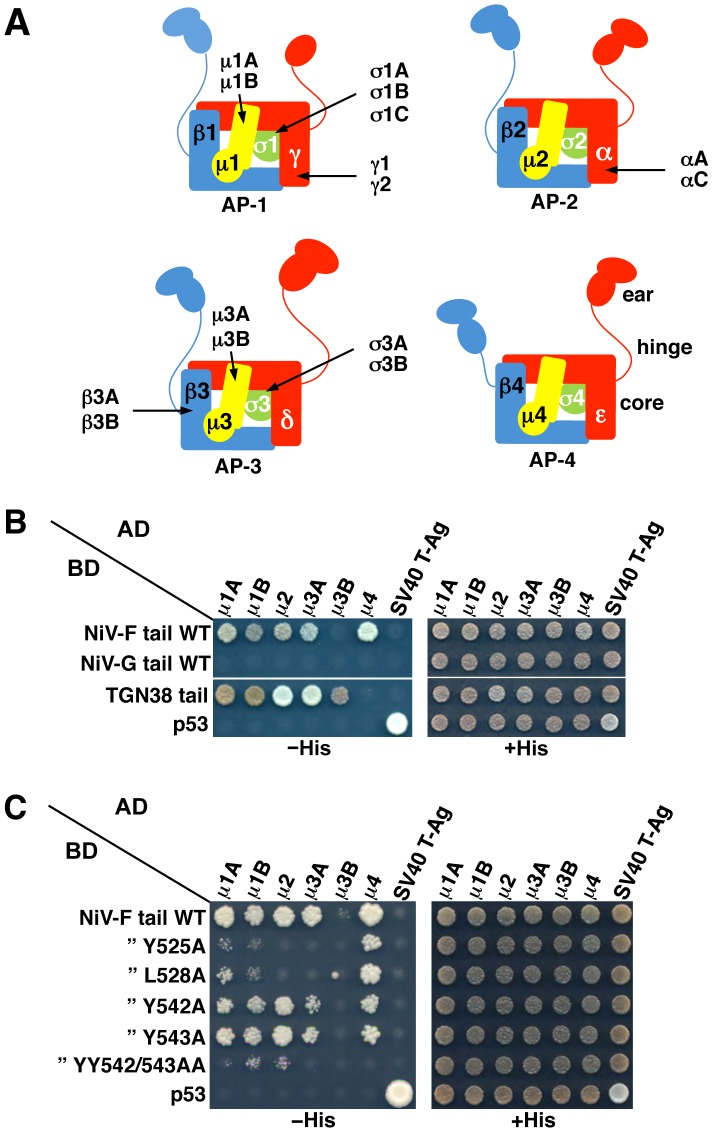
Interaction of the NiV-F cytosolic tail with AP μ subunits. (A) Scheme of heterotetrameric adaptor protein (AP) complexes depicting the four subunits in each complex along with subunit isoforms. Combinatorial assembly of subunits can originate multiple forms of AP-1, AP-2 and AP-3 [56]. (B) Y2H analysis showing interaction of the NiV-F cytosolic tail, but not the NiV-G cytosolic tail, with μ1A, μ1B, μ2, μ3A and μ4. Growth of yeast co-transformants on −His plates is indicative of interactions between tail constructs subcloned in a Gal4 binding domain (BD) vector and μ subunits subcloned in a Gal4 activation domain (AD) vector; growth on +His plates is a control for growth/loading of co-transformants. The TGN38 cytosolic tail was used as a positive control for interaction with various μ subunits. Co-transformations of cytosolic tail constructs with SV40 T-Ag and of μ subunits with p53 were used as negative controls. Co-transformation of p53 and SV40 T-Ag constructs provided an additional positive control for interactions. Images are composites of panels from the same experiments and are representative of three independent experiments. (C) Y2H analysis showing that alanine substitution of Y525 or L528 in the YXXØ-based signal or combined substitution of Y542 and Y543 inhibits the interaction of the NiV-F cytosolic tail with μ subunits. Experiments were performed as in panel B.

YXXØ signals are generally recognized by the μ1, μ2, μ3 and μ4 subunits of the heterotetrameric adaptor protein (AP) complexes AP-1, AP-2, AP-3 and AP-4, respectively (both μ1 and μ3 occur as A and B isoforms) [33], [34] (Figure 3A). Yeast two-hybrid (Y2H) analysis showed that the NiV-F cytosolic tail interacted with all the μ subunits tested, with the exception of μ3B (Figure 3B). In contrast, the NiV-G cytosolic tail, which lacks a YXXØ motif (Figure 1A), did not interact with any of the μ subunits (Figure 3B). Alanine substitution of Y525 or L528, corresponding to the Y and Ø positions of the YXXØ motif (Figure 1A), caused a drastic reduction in the interaction of the NiV-F tail with μ1A, μ1B, μ2 and μ3A, and to lesser extent, μ4 (Figure 3C). On the other hand, single substitution of Y542 and Y543 (Figure 1A) by alanine caused a partial reduction and double substitution an almost complete inhibition of interaction with all μ subunits (Figure 3C). These interactions correlated well with the function of the YXXØ motif, and to a lesser extent the YY motif, in somatodendritic sorting of NiV-F.

**Figure 4:**
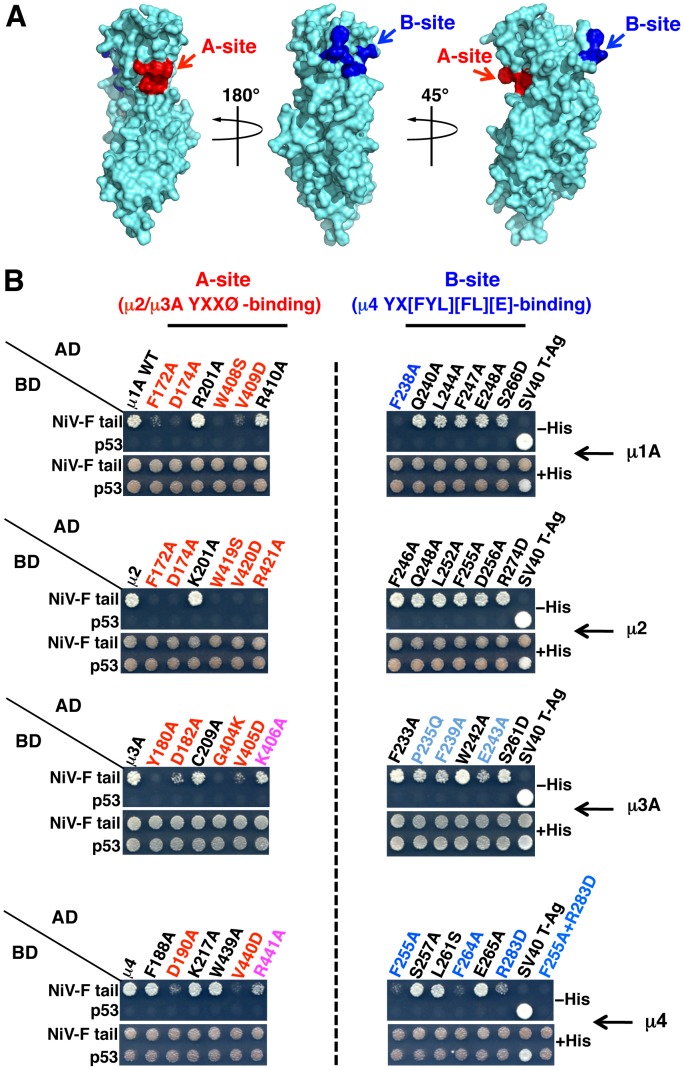
Binding sites on μ subunits involved in interactions with the NiV-F tail. (A) Surface representation of the three-dimensional structure of the μ1A C-terminal domain (residues 157–423) (PDB ID 1W63; [57]) exhibiting potential binding sites for YXXØ (A-site, red) and YX[FYL][FL][E] (B-site, blue) signals on opposite sides of the molecule. The potential A-site in μ1A was mapped by sequence alignment with μ2 and μ3A, and based on the structure of the complexes between the C-terminal domains of μ2 or μ3A and YXXØ signal-containing peptides [37], [38]. Mapping of the potential B-site in μ1A was based on sequence alignment with μ4 and on the structure of the C-terminal domain of this subunit in complex with a YKFFE-containing peptide [39]. Model images were built with PyMOL [58]. (B) Effect of alanine substitutions in critical residues of μ subunits A- and B-sites on the interaction with the NiV-F cytosolic tail. Residues in the putative A-sites of μ1A and μ4 were defined by homology with the residues in the corresponding sites of μ2 and μ3A [37], [38]. Similarly, residues in the putative B-sites of μ1A, μ2 and μ3A were defined by homology with those in the cognate site of μ4 [39]. Numbering of μ2 residues corresponds to the variant containing 433 residues (NCBI Reference Sequence NP_001020376). Residues whose substitution resulted in a strong inhibition in NiV-F tail binding are labeled in red (A-site) or dark blue (B-sites); substitutions causing a weaker inhibition are labeled in magenta (A-site) or light blue (B-site). Details of the Y2H analysis are as in the legend to Figure 3 B–C.

Structural studies have identified two distinct bindings sites for tyrosine-based sorting signals on the C-terminal domain of μ subunits. The first site, hereinafter termed A, is present on μ2 and μ3A and binds canonical YXXØ motifs from the EGFR and TGN38 cytosolic tails (YRAL and YQRL), respectively [37], [38]. The second site, termed B, is present on μ4 and binds a YX[FYL][FL][E] motif (a subset of YXXØ motifs) from the cytosolic tail of the amyloid precursor protein (YKFFE) [39]. Notably, the A- and B-sites lay on opposite faces (Figure 4A) and are predicted to be at least partially conserved in all μ subunits [38]. To test if these binding sites are involved in interactions with the NiV-F tail, we introduced alanine substitutions of key residues in μ1A, μ2, μ3A and μ4, and determined their effect using Y2H assays (μ1B was not analyzed because it is not expressed in neurons [40]). We found that substitution of several A-site residues in all the μ subunits abrogated binding to the NiV-F tail (Figure 4B, left panels). Substitution of B-site residues had less of an effect on binding to μ1A, μ2 and μ3A (only the F238A mutation in μ1A abolished binding), but caused substantial inhibition of binding to μ4 (Figure 4B, right panels). From these experiments, we concluded that binding of the NiV-F tail involves the A-site on μ1A, μ2 and μ3A, and both the A- and B-sites on μ4.

### Role of μ subunits in somatodendritic sorting of NiV-F

The experiments described above demonstrated that the NiV-F tail interacts with the μ1A, μ2, μ3A and μ4 subunits of the corresponding AP complexes. But, which of these interactions are responsible for the somatodendritic sorting of NiV-F? To address this question, we devised a dominant-negative approach in which neurons were transfected with plasmids encoding μ subunit A- or B-site mutants that are incapable of binding the NiV-F tail. Based on the Y2H analyses (Figure 4B), we selected the following single and double substitutions: μ1A D174A/W408S (A-site), μ2 D174A/W419S (A-site), μ3A D182A (A-site), μ4 D190A/V440D (A-site), and μ4 F255A/R283D (B-site). All constructs contained triple HA tags appended at their C-termini through a 10-amino acid linker [41] (Figure S1A). We demonstrated that the wild-type (WT) and mutant HA-tagged μ subunits were incorporated into their cognate AP heterotetramers, as shown by expression in HeLa cells followed by immunoprecipitation with anti-HA antiserum and immunoblotting for other subunits of the corresponding complexes (γ, α, β3 and ε for AP-1, AP-2, AP-3 and AP-4, respectively) (Figure S1B).

**Figure 5:**
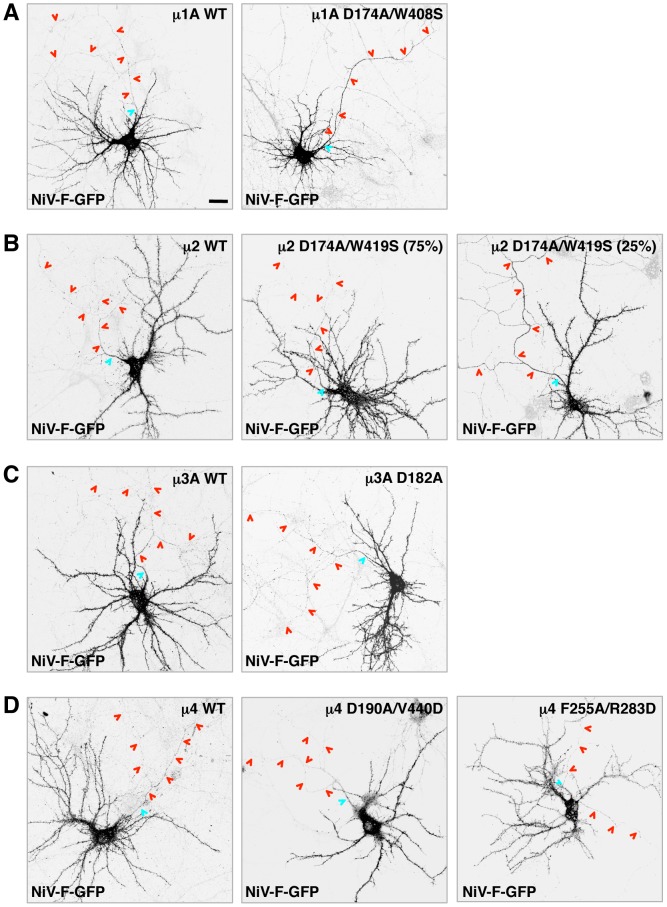
Effect of dominant-negative mutants of μ subunits on somatodendritic sorting of NiV-F. (A–D) Rat hippocampal neurons were co-transfected with plasmids encoding NiV-F-GFP and wild-type (WT) or dominant-negative (DN) mutants of HA-tagged μ subunits (A-site mutants of μ1A, μ2, μ3A and μ4, and B-site mutant of μ4, as labeled on top of images). The effects of μ1A, μ2, μ3A and μ4 constructs are shown in panels A, B, C and D, respectively. Cells were immunostained with mouse anti-HA, rabbit anti-MAP2 and goat anti-ANK-G, and imaged as indicated in the legend to Figure 1. Grayscale images shown in all panels correspond to NiV-GFP fluorescence. The anti-HA, anti-MAP2 and anti-ANK-G staining is shown in Figure S2. Two images corresponding to different effects on NiV-F-GFP sorting (representing ∼75 and 25% of the neuronal population) observed following expression of μ2 D174A/W419S are shown in (B). The AIS and axons are marked by cyan and red arrowheads, respectively. Scale bar: 20 µm. See Table 1 for polarity indexes.

Both the WT and D174A/W408S μ1A constructs exhibited staining of a juxtanuclear structure in the soma (Figure S2A), consistent with localization of AP-1 to the *trans*-Golgi network (TGN). Importantly, while expression of WT μ1A did not affect the somatodendritic localization of NiV-F-GFP (Figure 5A) (D/A: 7.9±2.0, as compared to 8.3±2.0 for NiV-F-GFP alone; Table 1), expression of the D174A/W408S A-site mutant resulted in non-polarized distribution of NiV-F-GFP (Figure 5A) (D/A: 1.6±0.4; Table 1). This indicated that interaction of the NiV-F tail with the A-site on μ1A is critical for its somatodendritic sorting. The observations in neurons expressing μ2 constructs were also of interest. The μ2 constructs localized to both dendrites and axons (Figure S2B). While expression of μ2 WT did not affect somatodendritic sorting of NiV-F (Figure 5B), expression of the D174A/W419S A-site mutant yielded a mixed phenotype: no effect in the majority of cells (∼75%) and non-polarized distribution in a minority of cells (∼25%) (Figure 5B). This heterogenous phenotype resulted in an average D/A of 5.3±3.1 (Table 1). In control experiments performed under the same transfection conditions, co-expression of μ2 D174A/W419S significantly increased surface levels of TfR-GFP in ∼70% of neurons compared to cells co-transfected with μ2 WT (Figure S3). This indicated that the heterogenous effect of μ2 D174A/W419S on NiV-F sorting in hippocampal neurons is not the result of lack of dominant negative activity of this mutant. The μ3A constructs were present in vesicles in both the somatodendritic and axonal domains whereas μ4 localized to the soma (Figure S2). Importantly, expression of WT μ3A or μ4, or mutants of these proteins that do not bind the NiV-F cytosolic tail, did not affect the somatodendritic sorting of NiV-F (Figure 5 C–D). These observations thus demonstrated a critical role for AP-1 in somatodendritic sorting of NiV-F through recognition of tyrosine-based signals by binding site A. AP-2 was important in only a subpopulation of cells, and AP-3 and AP-4 did not appear to be involved in this process.

### Assembly with NiV-G abolishes somatodendritic sorting of NiV-F

**Figure 6:**
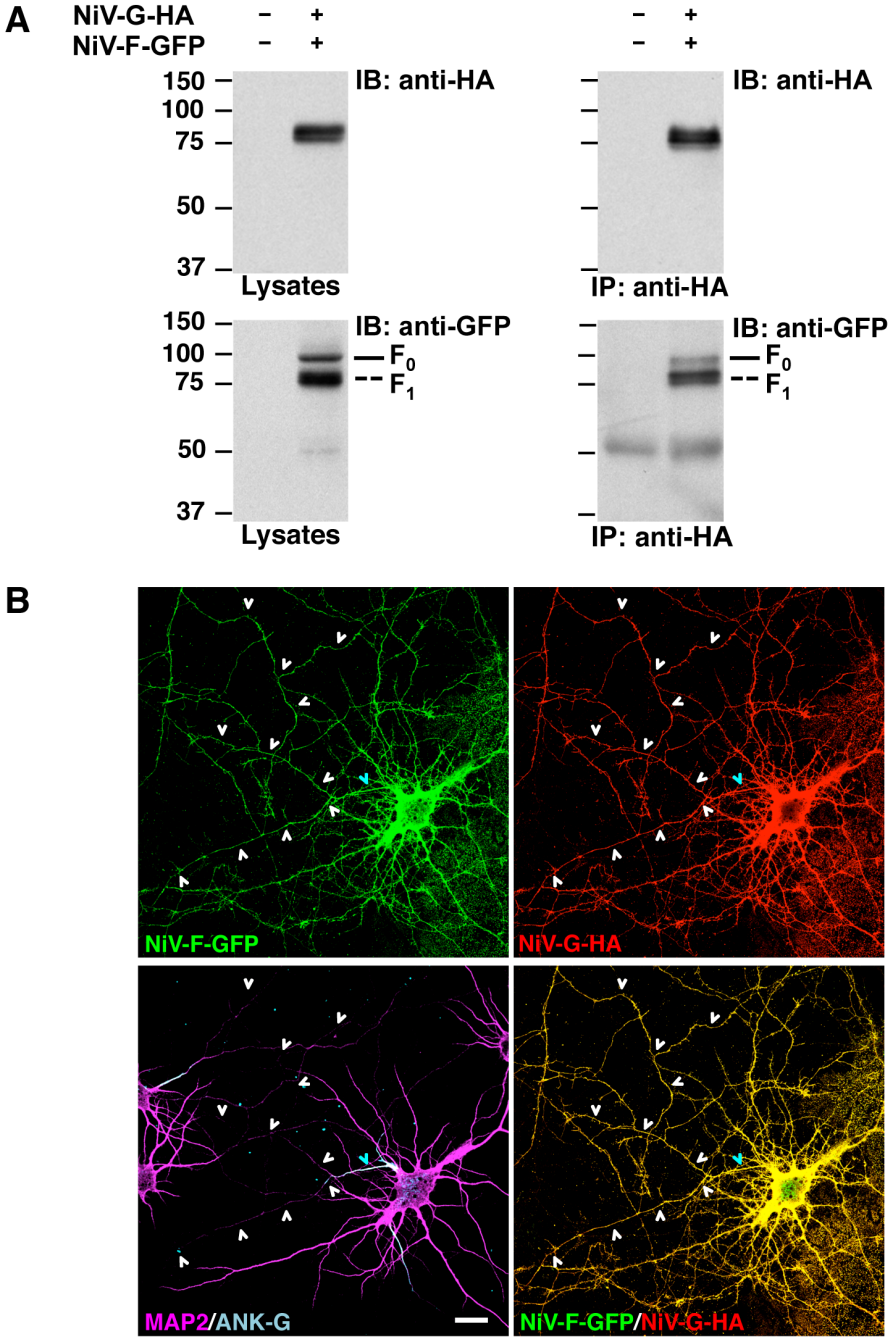
Interaction with NiV-G abolishes somatodendritic sorting of NiV-F. (A) Co-immunoprecipitation of NiV-F and NiV-G. HEK293T cells were co-transfected with NiV-F-GFP and NiV-G-HA; approximately 24–27 h post-transfection, cells were lysed and subjected to immunoprecipitation (IP) with rabbit anti-HA. Antigen-antibody complexes were eluted from beads and subjected to SDS-PAGE under reducing conditions and immunoblotting (IB) with either mouse anti-HA or mouse anti-GFP (upper and lower panels at right, respectively). Samples of cell lysates were also subjected to SDS-PAGE and immunoblotting (left panels). Full and dashed lines at right of bottom panels show the mobility of the NiV-F_0_ and -F_1_ forms (see Figure S4 for cleavage scheme). Co-IP of the NiV-F_2_ fragment with NiV-G could not be evaluated because the GFP moiety is fused to the C-terminus of NiV-F. The position of molecular mass markers (in kDa) is shown at left of blots. (B) Rat hippocampal neurons were co-transfected with NiV-F-GFP and NiV-G-HA, immunostained with mouse anti-HA, rabbit anti-MAP2 and goat anti-ANK-G, and imaged by confocal microscopy. Upper panels show NiV-F-GFP fluorescence (left) and anti-HA staining (right). The left lower panel shows anti-MAP2 (magenta) and anti-ANK-G (cyan) staining; the right lower panel displays merged images of NiV-F-GFP fluorescence (green) and anti-HA immunostaining (red) (yellow indicates co-localization). Cyan and white arrowheads show the position of the AIS and axons, respectively. Scale bar: 20 µm. Co-transfection with NiV-F and NiV-G constructs resulted in the frequent appearance of fused neurons (typically 2–3 cells) consistent with the role of these glycoproteins in cell-to-cell fusion [15], [26], [27]. Only isolated neurons co-transfected with NiV-F and NiV-G, showing unaltered distribution of MAP2 and ANK-G markers, were selected for quantitative analysis of polarity of the two glycoproteins (Table 1).

Given that interactions between fusion and attachment proteins of paramyxoviruses are critical for host cell invasion [22], we hypothesized that NiV-F and NiV-G might mutually influence their polarized sorting in neurons. We tested this hypothesis in rat hippocampal neurons co-transfected with plasmids encoding tagged forms of NiV-F and NiV-G. Co-immunoprecipitation experiments in HEK293T cells showed that NiV-G-HA interacted with both the uncleaved F_o_ (fusion-inactive) and cleaved F_1_ (fusion-active) forms (Figure 1A) of NiV-F-GFP (Figure 6A). Importantly, co-transfection of neurons with NiV-G-HA resulted in non-polarized distribution of NiV-F-GFP (Figure 6B) (D/A: 1.3±0.2; Table 1), in sharp contrast with the somatodendritic sorting observed following single expression of NiV-F-GFP (D/A: 8.3±2; Figure 1B and Table 1). Conversely, the non-polarized localization of NiV-G-HA to both the somatodendritic and axonal domains was not affected by co-expression of NiV-F-GFP (D/A: 1.2±0.4 and 1.0±0.2 for NiV-G-HA in the presence or absence of NiV-F-GFP, respectively; Table 1). These experiments thus demonstrated that assembly with NiV-G abolished the polarized, somatodendritic distribution of NiV-F.

Newly synthesized NiV-F is transported as the inactive F_0_ form to the plasma membrane; following endocytosis, F_0_ is cleaved in endosomes to the active disulfide-linked F_2_-F_1_ form (Figure S4A), which recycles back to the plasma membrane [20]. Given the relationship between NiV-F activity and cellular localization, we asked whether the polarized sorting of NiV-F and the regulatory effect of NiV-G depended on NiV-F activity. To this end, we generated a mutant form of NiV-F-GFP having a deletion in the cleavage site (NiV-F-Δ104–109-GFP) [42]. This mutant displayed a marked reduction in proteolysis as observed upon expression in HEK293T cells (Figure S4B), but exhibited somatodendritic sorting in neurons (D/A: 7.5±2.0), similar to that of the wild-type protein (D/A: 8.3±2.0) (Figure S4C; Table 1). Furthermore, co-expression of NiV-G-HA resulted in non-polarized distribution of NiV-F-Δ104–109-GFP (Figure S4D) (D/A: 1.2±0.3, Table 1), similar to the observations with NiV-F WT. Therefore, somatodendritic sorting of NiV-F and its depolarization by NiV-G are not dependent on proteolytic activation of NiV-F.

We also analyzed whether inhibition of AP-2-dependent endocytosis affected the ability of NiV-G to depolarize NiV-F. Experiments were carried out in neurons subjected to triple transfection for 24 or 48 h with NiV-F-GFP, NiV-G-mCherry and either μ2 WT or the μ2 D174A/W419S dominant negative mutant. As a control, we measured the effects of μ2 D174A/W419S on surface levels of TfR under these transfection conditions. Although expression of μ2 D174A/W419S enhanced surface labeling of TfR in neurons transfected for 24 or 48 h, it did not affect the depolarization of NiV-F caused by co-expression of NiV-G (Figure S5) (D/A: 1.4±0.3 and 1.5±0.4 for cells co-transfected with WT or dominant negative μ2, respectively, Table 1), indicating that this effect does not depend on AP-2 dependent endocytosis.

### Expression of NiV-G promotes axonal transport of NiV-F

**Figure 7:**
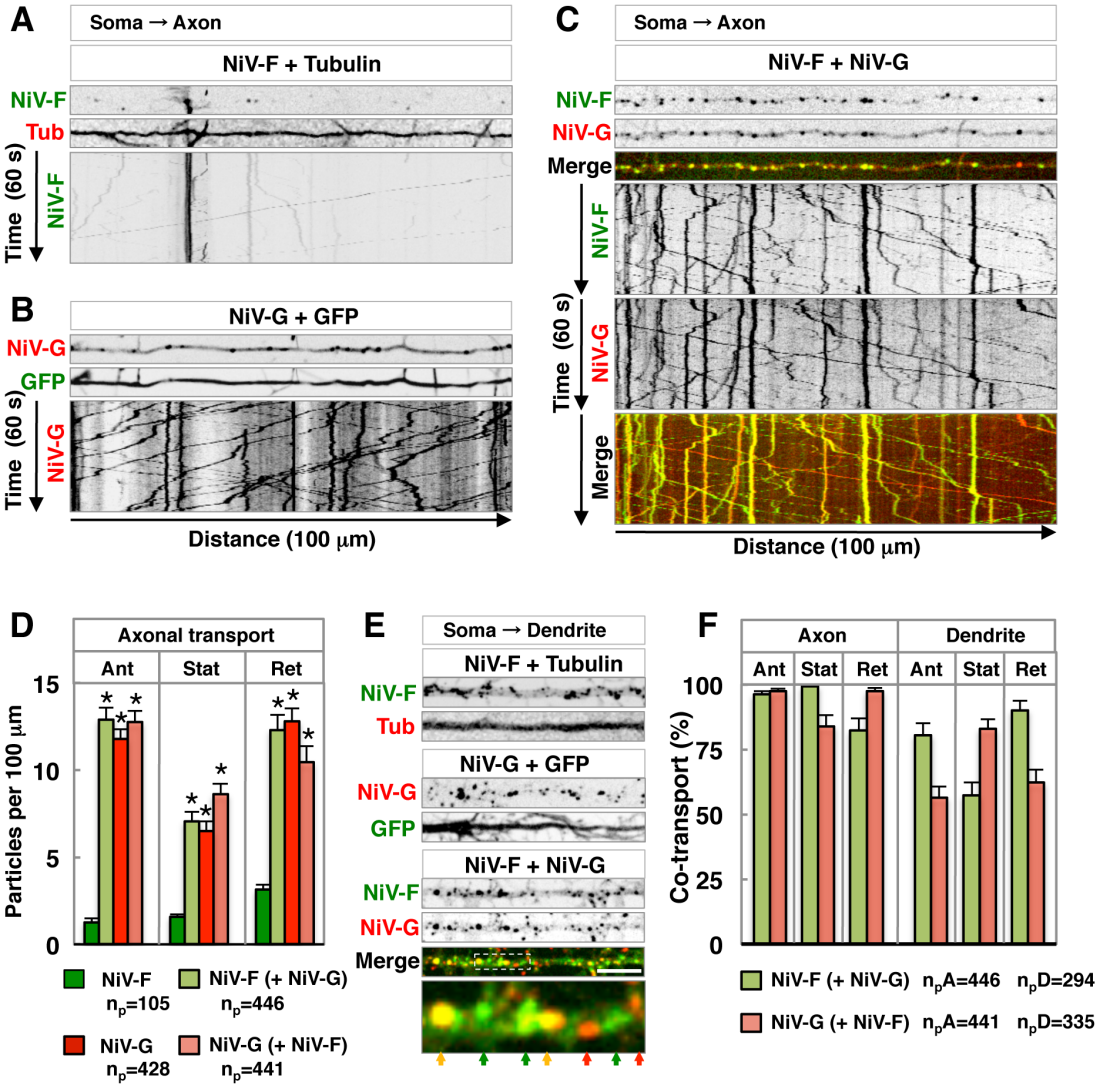
Live-cell imaging shows that NiV-G increases axonal transport of NiV-F. (A–C) Single-frame images from Video S1 (thin upper images) and kymographs (bottom images) of the analysis of particles moving along 100 µm of axons in DIV10 rat hippocampal neurons co-transfected on DIV5 with plasmids encoding either NiV-F-GFP (NiV-F) and mCh-Tub (Tub) (panel A), NiV-G-mCh (NiV-G) and GFP (panel B) or NiV-F-GFP and NiV-G-mCh (panel C). Images in (A) and (B) are shown in grayscale. In panel (C), NiV-F-GFP and NiV-G-mCh fluorescence are shown individually in grayscale and as green and red, respectively, in merged images (yellow indicates co-localization). Tracings with negative and positive slopes in kymographs represent anterograde and retrograde movement of particles, respectively; vertical lines represent particles that are stationary during the 60 s recording. (D) Quantification of NiV-F-GFP and NiV-G-mCh axonal transport in neurons expressing these proteins individually or in combination. Data shown represent the number of anterograde (Ant), stationary (Stat) and retrograde (Ret) particles per 100 µm of axon length during the 60 s recording. NiV-F and NiV-G (green and red bars, respectively) show the number of axonal particles containing these two proteins when expressed individually. “NiV-F (+NiV-G)” (light green bars) is the number of NiV-F-GFP particles in neurons co-expressing NiV-G-mCh; “NiV-G (+NiV-F)” (salmon bars) represents the number of NiV-G-mCh particles in neurons co-expressing NiV-F-GFP. Values are the means±SEM of 20–22 independent measurements for each condition and represent the total number of particles (n_p_) indicated under the graph. Statistical significance was calculated by one-way ANOVA followed by Dunnett's test. (*) *P*<0.01 when compared to NiV-F-GFP-containing particles in cells expressing only this protein. (E) Single-frame images from Video S2 showing dendritic particles from neurons co-expressing either NiV-F-GFP and mCh-Tub, NiV-G-mCh and GFP, or NiV-F-GFP and NiV-G-mCh (upper panels in grayscale; bottom panel with merged NiV-F-GFP and NiV-G-mCh images in green and red, respectively). The lower color image is a 4× magnification of the boxed area in the above image; color arrows point to particles containing either NiV-F-GFP or NiV-G-mCh (green and red, respectively) or both proteins (yellow). Scale bar: 10 µm. (F) Co-localization of NiV-F-GFP and NiV-G-mCh in axonal and dendritic particles of co-transfected neurons. NiV-F-GFP co-localization in co-transfected neurons (NiV-F (+NiV-G), light green bars) was the percentage of NiV-F-GFP-containing particles that also contained NiV-G-mCh. Similarly, NiV-G-mCh co-localization (NiV-G (+NiV-F), salmon bars) was the percentage of NiV-G-mCh-containing particles that also displayed NiV-F-GFP. Values are the means±SEM of 22 and 33 measurements of axonal and dendritic particles, respectively, and represent the total number of axonal and dendritic particles (n_p_A and n_p_D) containing NiV-F-GFP and NiV-G-mCherry indicated under the graph.

The effect of NiV-G on NiV-F sorting was examined in more detail by live-cell imaging of transfected rat hippocampal neurons. We observed very little transport of NiV-F-GFP along the axon in the absence of NiV-G-mCherry (Figure 7A and Video S1). In contrast, numerous axonal carriers containing NiV-G-mCherry were observed in the absence of NiV-F-GFP (Figure 7B and Video S1). Co-expression of NiV-F-GFP with NiV-G-mCherry greatly increased the amount of NiV-F-GFP in both anterograde and retrograde axonal carriers, as well as in stationary foci (Figure 7 C–D; Video S1). The amount of NiV-G-mCherry in these structures, on the other hand, was not affected by co-expression with NiV-F-GFP (Figure.7 C–D; Video S1). Analysis of dendrites showed that both NiV-F-GFP and NiV-G-mCherry were transported in vesicular carriers irrespective of whether they were expressed singly or in combination (Figure 7E; Video S2). We concluded that NiV-G abolishes polarized sorting of NiV-F by promoting incorporation of NiV-F into axonal transport carriers at the level of the soma.

We quantified the axonal and dendritic vesicular structures (anterograde and retrograde carriers, and stationary foci) that contained both NiV-F-GFP and NiV-G-mCherry in co-expressing live cells. We observed a higher proportion of anterograde carriers containing both glycoproteins in axons (96–97%) as compared to dendrites (56–80%), consistent with the notion that NiV-G is required for incorporation of NiV-F into axonal transport carriers and axonal entry, likely as part of NiV-F•NiV-G complexes (Figure 7F). On the other hand, the presence of subpopulations of dendritic vesicles carrying only NiV-F or NiV-G suggests that their somatodendritic transport can occur independently of each other.

### Biosynthetic trafficking of NiV-F and NiV-G

**Figure 8:**
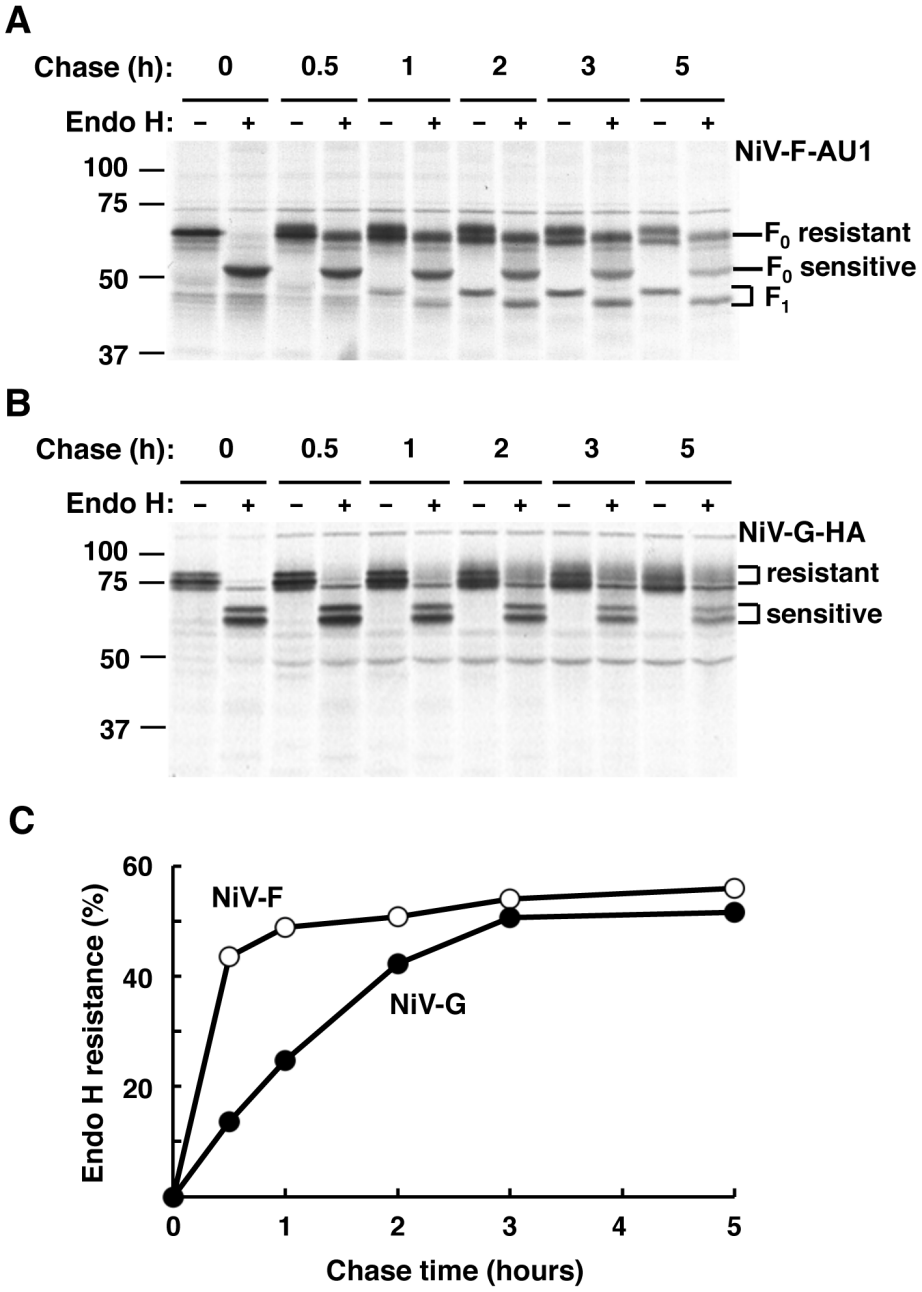
NiV-F and NiV-G exhibit different rates of biosynthetic trafficking. HEK293T cells transfected with NiV-F-AU1 (A) or NiV-G-HA (B) were pulse-labeled for 15 min and chased for the indicated times; this was followed by lysis, immunoprecipitation with anti-epitope antibodies and treatment in the presence or absence of endo H. Samples were subjected to SDS-PAGE under reducing conditions and autoradiography. (A) NiV-F_0_ displays four utilized sites of N-linked glycosylation [26], [27] (Figure 1A) some of which become endo H-resistant while others remain endo H-sensitive upon transport through the Golgi complex. Golgi processing is evidenced by the appearance of a lower mobility band from 0.5 h of chase onwards in lanes with undigested immunoprecipitates. Treatment of the Golgi-processed form of NiV-F_0_ with endo H results in an intermediate mobility shift (apparent from 0.5 h of chase onwards) referred to as “F_0_ resistant”, while digestion of the unprocessed form results in a larger shift referred to as “F_0_ sensitive” (visible from time 0 of chase onwards) (corresponding lines at right of panel). The NiV-F_1_ form, whose production depends on transport of newly synthesized NiV-F_0_ to the plasma membrane followed by endocytosis and cleavage in acidic endosomes [20], [29], [30], was apparent after 1 h of chase and exhibited partial resistance to endo H (bands indicated by bracket at right of panel). The NiV-F_2_ fragment was not detected because the AU1 epitope is located at the C-terminus of the tagged protein (see Figure S4A for NiV-F cleavage scheme). (B) NiV-G displays six utilized sites of N-linked glycosylation [28] (Figure 1A). The position of endo H-resistant and -sensitive forms of NiV-G is shown by brackets. Note the slower production of endo H-resistant NiV-G compared to that of endo H-resistant NiV-F_0_ shown in panel A. Data in panels A and B are representative of two independent experiments where NiV-F-AU1- and NiV-G-HA-transfected cells were pulsed, chased and processed simultaneously. The position of molecular mass markers (in kDa) is shown at left. (C) Densitometric analysis of autoradiograms in panels A and B was performed with Image J software (http://rsbweb.nih.gov/ij). Results shown represent the percentage of endo H resistance (100×endo H resistant forms/endo H resistant forms+endo H sensitive forms) at different times of chase (only the F_0_ form was considered in the quantitative analysis of NiV-F).

We also asked whether the observed differences in sorting of NiV-F in the presence or absence of NiV-G occur in the context of differences in biosynthetic trafficking that may transiently separate these glycoproteins. We examined the rates of biosynthetic trafficking in HEK293T cells transfected with NiV-F-AU1 or NiV-G-HA and subjected to pulse-chase metabolic labeling followed by immunoprecipitation and endoglycosidase H (endo H) treatment. Consistent with studies on the glycoproteins of the related Hendra virus [43], our results showed that newly synthesized NiV-F achieves maximum endo H resistance, a measure of transit through the Golgi complex, at ∼0.5–1 h of chase, while NiV-G reaches a similar level of processing at 2–3 h of chase (Figure 8). Production of NiV-F_1_, which depends on transport of newly synthesized NiV-F_0_ to the plasma membrane followed by endocytosis and cleavage in acidic endosomes [20], [29], [30], was apparent after 1 h of chase consistent with the faster biosynthetic trafficking of NiV-F (Figure 8A).

## Discussion

### Polarized sorting of NiV-F and NiV-G in neurons

Our studies demonstrate that, when expressed individually, NiV-F is sorted to the somatodendritic domain, whereas NiV-G exhibits a non-polarized distribution in hippocampal neurons. Previous studies identified various sequence motifs in the cytosolic tails of transmembrane proteins that mediate somatodendritic sorting [35], [36], [44]–[46]. Here we show that somatodendritic sorting of NiV-F is dependent on a canonical YXXØ motif (residues 525–528) and, less markedly, on a di-tyrosine motif (residues 542–543) in its cytosolic tail. Although the NiV-G tail also contains a di-tyrosine motif (residues 28–29; Figure 1A), the lack of a YXXØ motif is presumably responsible for the non-polarized distribution of this protein in hippocampal neurons.

The neuronal sorting of NiV-F and NiV-G exhibits similarities and differences when compared to their basolateral sorting in epithelial cells [31] and their bipolar distribution in endothelial cells [32]. The basolateral distribution of NiV-F in polarized MDCK epithelial and microvascular endothelial cells depends on its YXXØ motif; however, only basolateral sorting in endothelial cells is altered by substitution of the NiV-F di-tyrosine sequence [31], [32]. On the other hand, substitution of the di-tyrosine sequence in the NiV-G cytosolic tail impairs basolateral sorting of this glycoprotein in MDCK but not in microvascular endothelial cells [31], [32]. Moreover, the non-polarized distribution of NiV-G in neurons is in sharp contrast with its basolateral localization in epithelial cells [31]. These observations demonstrate that various cell types interpret sorting information differently, and highlight additional variations to the analogy between somatodendritic and axonal domains in neurons and basolateral and apical domains in other polarized cell types, respectively [1], [6].

Importantly, we have identified the mechanism responsible for somatodendritic sorting of NiV-F through a combined approach including Y2H assays of its interactions with AP μ subunits and co-transfection of neurons with NiV-F and dominant-negative mutants of μ subunits. Y2H assays revealed that the NiV-F cytosolic tail interacts with the μ subunits of AP-1, AP-2, AP-3 and AP-4 in a manner dependent on both the YXXØ and di-tyrosine motifs present in this tail. This dependence on two tyrosine-based motifs suggests that the NiV-F tail might simultaneously interact with both potential binding sites (A and B) on μ subunits. This is consistent with the distance between the YXXØ (residues 525–528) and the di-tyrosine (residues 542–543) motifs (14 residues corresponding to ∼45 Å in an unstructured peptide) and the 30 Å separation between the A-site and B-site on the overlapped structures of μ2 and μ4 [39]. It is also possible that the di-tyrosine plays a conformational role, uncovering and/or stabilizing the YXXØ motif in the NiV-F tail for recognition by the various μ subunits.

The identification of residues required for binding of the NiV-F tail allowed us to design μ-subunit constructs that act as dominant-negative mutants by interfering with the endogenous AP complexes involved in somatodendritic sorting of NiV-F. This analysis conclusively demonstrated that the clathrin adaptor AP-1 plays a critical role in the targeting of NiV-F to the somatodendritic domain. This is consistent with the role of AP-1 in the somatodendritic sorting of other proteins including TfR, the metabotropic glutamate receptor mGluR1 and the NMDA receptor subunits NR2A and NR2B [36]. We also detected a non-polarized distribution of NiV-F in ∼25% of hippocampal neurons transfected with the μ2 A-site mutant. Hence, together with AP-1, AP-2 may also play a role in somatodendritic sorting of NiV-F in a subpopulation of hippocampal neurons. Although we have not addressed how AP-2 affects NiV-F sorting, the involvement of this adaptor is consistent with the idea that both biosynthetic and endocytic pathways contribute to neuronal protein sorting [1].

Our observations thus demonstrate that viral membrane proteins such as NiV-F, with tyrosine-based signals in their cytosolic tails, utilize the same neuronal machinery that is responsible for somatodendritic sorting of endogenous proteins. At the same time, they support the proposed role of AP-1 as a general regulator of polarized sorting [36], [47].

### Regulation of NiV-F sorting by NiV-G

Our observations shed light on another important layer of regulation in the neuronal sorting of viral envelope glycoproteins that interplays with the machinery responsible for polarized distribution of endogenous proteins. Whereas single transfection of neurons with NiV-F resulted in its somatodendritic sorting, co-expression of NiV-G caused non-polarized distribution of NiV-F. This effect was analyzed not only by confocal microscopy but also by live-cell imaging, allowing us to observe axonal and dendritic transport vesicles carrying both NiV-F and NiV-G in cells co-expressing the two envelope proteins.

Previous studies addressed the effects of co-expression of NiV glycoproteins on their sorting in epithelial cells. Although both NiV-F and NiV-G are localized basolaterally when expressed individually in polarized MDCK cells, co-expression results in non-polarized distribution of the two proteins [31]. Studies with NiV-F mutants showed that co-sorting with NiV-G may result from intrinsic membrane fusion activity of the NiV-F•NiV-G complex that affects the integrity or polarity of infected epithelial cells [15], [31]. Our observations point to another difference in the sorting of NiV glycoproteins in epithelial cells and neurons. The NiV-F-Δ104–109 mutant displays impaired proteolytic activation, but is sorted to the somatodendritic domain of hippocampal neurons in a manner similar to that of the wild-type protein. Importantly, co-expression of NiV-G also results in non-polarized distribution of NiV-F-Δ104–109, indicating that this effect is unrelated to microfusion events that can alter neuronal polarity. This finding is also consistent with the unaltered distribution of the somatodendritic marker MAP2 in neurons co-expressing the two NiV glycoproteins. Consequently, the most likely mechanism underlying the non-polarized distribution of NiV-F following co-expression of NiV-G is that formation of NiV-F•NiV-G complexes interferes with the interaction between NiV-F and AP-1 and, consequently, with exclusion of NiV-F from axonal transport carriers.

There are precedents for viral proteins driving the redistribution of other viral components in epithelial cells. For instance, the Marburg virus matrix protein VP40 triggers the re-localization of the glycoprotein GP from the apical to the basolateral membrane of epithelial cells, leading to budding of infectious virions from epithelial cells [13]. Interactions of viral proteins with cytoskeleton components of peripheral nervous system (PNS) neurons that facilitate virus spread have also been reported. Us9 is a conserved membrane protein encoded by alphaherpesviruses that interacts with the microtubule-dependent kinesin-3 motor KIF1A to regulate anterograde axonal transport of other viral proteins and viral particles [48]. Our observations extend these precedents and uncover a novel mechanism of regulation of neuronal sorting exclusively involving the two envelope glycoproteins encoded by a central nervous system (CNS)-targeting virus.

### Possible role of NiV-G-dependent regulation of NiV-F sorting in neurons

**Figure 9:**
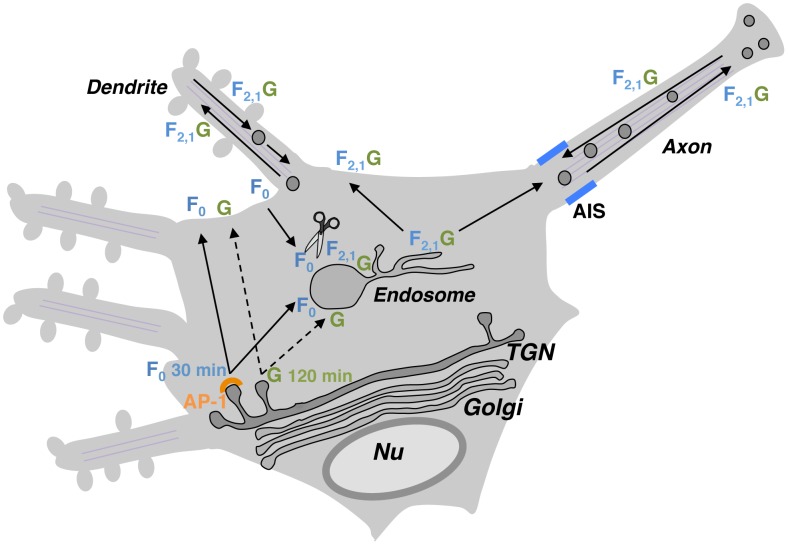
Proposed regulation of NiV-F neuronal sorting by NiV-G. NiV-F (F_0_ inactive precursor) and NiV-G (G) exhibit relatively fast and slow biosynthetic trafficking, respectively (full and dashed lines in scheme). In the absence of G, F_0_ is first sorted to the somatodendritic plasma membrane and then internalized to early/recycling endosomes located in the soma where it undergoes proteolytic activation (generation of F_2,1_). This allows for efficient activation of NiV-F within the somatodendritic domain. The interaction between active F_2,1_ and newly synthesized G (exhibiting delayed trafficking out of the TGN) alters the sorting of the fusion glycoprotein and results in the presence of NiV-F•NiV-G complexes (F_2,1_G) not only in dendrites but also in the axonal compartment. Although depolarization of NiV-F by NiV-G may also occur when AP-2-dependent endocytosis is affected (Figure S5), the endocytosis-dependent proteolytic activation of NiV-F [20], [29], [30] and the different rates of biosynthetic trafficking makes endosomes the most plausible site of formation of NiV-F•NiV-G complexes. Localization of NiV-F•NiV-G complexes to axons may facilitate transneuronal spread of NiV. Differential sorting of NiV-F in the presence or absence of NiV-G may coordinate NiV-F processing, axonal transport and anterograde spread between neurons.

Our observations establish that NiV envelope glycoproteins can regulate their own neuronal sorting. Specifically, formation of a complex with NiV-G allows NiV-F to escape the somatodendritic restriction otherwise imposed by an AP-1-dependent mechanism. What is the advantage conferred by this regulation? We have shown that the biosynthetic transport of NiV-G is much slower (∼120 min) compared to that of NiV-F (∼30 min). This difference in biosynthetic transport rate, along with the required endocytosis-dependent proteolysis of NiV-F, imposes time and spatial constraints for the formation of active NiV-F•NiV-G complexes [22]. We propose that the interplay among (a) differences in biosynthetic transport of NiV-F and NiV-G, (b) the AP-1-dependent-targeting of NiV-F to the somatodendritic plasma membrane, (c) its endocytosis and proteolytic activation in endosomes and (d) the subsequent formation of NiV-F•NiV-G complexes, constitutes a highly coordinated and efficient system for delivering active envelope glycoproteins to the entire neuronal surface (Figure 9). In this model, the initial sorting of NiV-F to the somatodendritic plasma membrane, preceding NiV-G, ensures the subsequent endocytosis to endosomes that are abundant in the somatodendritic compartment [1]. Following proteolysis catalyzed by cathepsin L or B, the active NiV F_2_-F_1_ assembles with the slower-trafficking NiV-G resulting in a non-polarized distribution of the complex along both the somatodendritic and axonal compartments (Figure 9).

It is not known at this time to what extent NiV spreads in the CNS by release of infectious particles, syncytium formation or microfusion events at synaptic contacts. Similarly, no studies have addressed whether NiV spreads interneuronally in an anterograde or retrograde manner. Nonetheless, it has been proposed that interneuronal spread along neuronal pathways may contribute to NiV dissemination [18], and that axonal transport may contribute to NiV spread across the CNS [19]. These findings are consistent with the localization of NiV-G-binding ephrin-B ligands to post-synaptic densities of mouse hippocampal CA1 neurons [49], and with our observations revealing co-localization of ephrin-B3 and the post-synaptic membrane marker post-synaptic density protein 95 in rat hippocampal neurons (unpublished observations). Although the specific mode of NiV spread is a critical issue that deserves further study, it seems reasonable to conclude that the interregulation of NiV glycoprotein sorting and ensuing accumulation of NiV-F•NiV-G complexes in the somatodendritic and axonal domains may represent a key mechanism to increase the likelihood of viral spread through synaptic contacts.

## Materials and Methods

### Ethics statement

Animal work was conducted following the guidelines established by the National Institute of Child Health and Human Development (NICHD), Animal Care and Use Committee (ACUC). Research was performed under protocols #10-103 and #13-011 approved by the NICHD ACUC. The NICHD ACUC follows the United States regulations and guidelines set forth by the National Institutes of Health, the Animal Welfare Act, the Guide for the Care and Use of Laboratory Animals, the Public Health Service Policy on Humane Care and Use of Laboratory Animals and the Government Principles for the Utilization and Care of Vertebrate Animals used in Testing, Research and Training.

### DNA constructs

pcDNA3.1-NiV-F-AU1 and pcDNA3.1-NiV-G-HA containing codon-optimized cDNAs [27], were gifts from B. Lee (University of California at Los Angeles). cDNAs encoding the cytosolic tails of NiV-F and NiV-G were provided by A. Maisner (Philipps University of Marburg). The monomeric Cherry-tubulin construct, pEGFP-N1 (Clontech) with the EGFP dimer-interfering A206K mutation and pmCherry-N1 were gifts from J. Lippincott-Schwartz (NIH, Bethesda, MD). The pEGFPA206K-N1 human TfR construct was previously described [36].

An EcoRI/BamH1 fragment encoding NiV-F and an EcoRI/XhoI fragment encoding NiV-G were subcloned into the corresponding sites of pEGFPA206K-N1 and the EcoRI/SalI sites of pmCherry-N1, respectively. An XhoI/EcoRI fragment encoding the extracellular and transmembrane domains of human interleukin-2-receptor α subunit (referred to as Tac) (residues 1–261) and an EcoRI/XhoI fragment encoding NiV-F 519–546 (cytosolic tail) followed by a stop codon were subcloned in frame into the XhoI/SalI of pEGFP-N1. The pCI-neo-(Promega) based construct for the expression of mouse μ1A appended at the C-terminus with a 10-residue spacer GSGSGGSGSG and three copies of the HA epitope has been described [41]. An EcoRI/Sal I fragment encoding mouse μ1A plus the spacer and one HA copy was excised from this construct and replaced by corresponding fragments encoding mouse μ2, rat μ3A or human μ4. The μ1A, μ1B, μ2, μ3A and μ4 constructs in the Gal4-activation domain vector pACT2 (Clontech) have been described [41]. The cytosolic tails of NiV-F (residues 519–546) and NiV-G (residues 1–45) were subcloned in the Gal4-binding domain pGBKT7 (Clontech), while the rat TGN38 tail 324–353 fragment was subcloned into pGBT9 (Clontech) [50]. All mutations in NiV-F and μ subunits were generated by site-directed mutagenesis (QuickChange; Agilent).

### Antibodies

Rabbit anti-MAP2 and goat anti-ANK-G (P-20) antisera were from Santa Cruz Biotechnology. Rabbit anti-HA was a gift from Ajay Sharma (NIH, Bethesda, MD). The mouse anti-HA epitope and mouse anti-GFP antibodies were from Covance and Roche Applied Science, respectively, while the rabbit anti-GFP antiserum was from Invitrogen. The mouse anti-AP-1 γ, anti-AP-2 α and anti-AP-4 ε antibodies were from BD Biosciences. The rabbit anti-AP-3 β3 antiserum (β3H7) was previously described [51].

### Cell culture and transfection

Primary hippocampal neuronal cultures were prepared as previously described [52] and maintained at 37°C under a humidified atmosphere (95∶5 air∶CO_2_). Briefly, hippocampi were dissected from Sprague Dawley rats on embryonic day 18. Dissociated cells were plated onto poly-L-lysine- and laminin-treated plates and maintained in Dulbecco's modified Eagle's medium (DMEM) supplemented with 10% horse serum for 2–3 h. The medium was then substituted with Neurobasal medium supplemented with B-27 and Glutamax. After 4 days in culture (DIV4), neurons were transfected with different constructs at a ratio of ∼0.5–1 µg DNA per 3×10^4^ cells using Lipofectamine 2000. Transfected neurons were analyzed at DIV10. HeLa and HEK293T cells were cultured on 100-mm or 150-mm dishes at 37°C under 95∶5 air∶CO_2_ in DMEM/high glucose supplemented with 10% (v/v) fetal bovine serum, 100 U/ml penicillin and 100 µg/ml streptomycin. Cells were transfected at ∼30–50% confluence with a total of 7–10 µg (100-mm plates) or 20 µg (150-mm plates) of DNA constructs per plate using the Fugene (Promega) or X-tremeGENE 9 (Roche) reagents.

### Immunofluorescence microscopy and image quantification

Neurons were fixed with 4% v/v paraformaldehyde and 4% w/v sucrose in phosphate-buffered saline (PBS) for 20 min, permeabilized with 0.2% v/v Triton X-100 for 15 min, blocked with 0.2% w/v gelatin for 30 min at 37°C and stained with primary antibodies overnight at 4°C, followed by secondary antibodies for 30 min at 37°C. MAP2 and ANK-G antibodies were used to identify the dendrites and the AIS, respectively. All fluorescence images were acquired using a confocal microscope (LSM710, Zeiss). For quantification, we used Image J version 1.44o (Wayne Rasband, NIH, http://imagej.nih.gov). For each condition, 8–14 cells from two or three different cultures were analyzed. The polarity index was quantified as described previously [53]. Briefly, several 1-pixel-wide lines were traced along 3 dendrites and representative portions of the axon using MAP2, GFP or mCh-tubulin staining as guides. An average dendrite/axon (D/A) ratio was calculated for each cell: D/A = 1, uniform staining; D/A>1, preferential dendritic staining; D/A<1, preferential axonal staining.

For TfR-GFP surface staining, transfected neurons were fixed and blocked as indicated above and incubated with rabbit anti-GFP antibody either overnight at 4°C or for 30 min at 37°C. At the end of this period, cells were washed three times with calcium- and magnesium-containing PBS, blocked, permeabilized and incubated with mouse anti-HA overnight at 4°C. Cells were subsequently incubated with Alexa 555-conjugated anti-rabbit and Alexa-405 conjugated anti-mouse antisera for 30 min at 37°C.

### Live-cell imaging

Neurons were transfected on DIV5 and imaged on DIV10 using a spinning disk microscope (Marianas; Intelligent Imaging) equipped with a 63×, 1.4 N.A. objective. Digital images were acquired with an EM-CCD camera (Evolve; Photometrics). NiV-F-GFP or NiV-G-mCh were exposed for 200 ms and recorded each 500 ms for 60 s. For dual-color videos, NiV-F-GFP and NiV-G-mCh were exposed for 200 ms sequentially and recorded each 500 ms for 60 s. Four transfected neurons from three different experiments were chosen for time-lapse imaging, and a single level of focus was maintained throughout each recording. Dendrites and axons were identified morphologically by their length and thickness, and also by co-transfection with mCh-tubulin or GFP (staining of both dendrites and axons) or Tau-CFP (staining of axons). Image processing and analysis were performed using Image J. Images of axons and dendrites were isolated, straightened and cropped to equal dimensions for direct comparison of transport parameters. Kymographs were generated by re-slicing stacks followed by Z-projection. Axons and dendrites were orientated so that anterograde movement occurred from left to right. Axonal vesicle movement was measured starting at 30–40 µm distance from the soma to exclude the AIS. The number of anterograde and retrograde vesicles moving or co-moving and stationary particles were determined manually from kymographs of axon and dendrites. We defined as stationary a particle that paused for the entire duration of the imaging period (60 s).

### Biochemical assays

Transfected HEK293T or HeLa cells in 100-mm plates were washed with PBS and lysed in 800 µl of 50 mM Tris-HCl pH 7.4, 0.8% (v/v) Triton X-100, 75 mM NaCl (lysis buffer) with protease inhibitors (EDTA-free Complete, Roche Applied Science). Extracts were centrifuged for 15 min at 21,000 g and 4°C. Supernatants were precleared by incubation with protein G-Sepharose beads (GE Healthcare), supplemented with 0.1% bovine serum albumin (BSA) and subjected to overnight immunoprecipitation with rabbit or mouse anti-HA immobilized onto the same beads. Beads with bound complexes were washed three times with lysis buffer containing 0.1% (v/v) Triton X-100 and once with PBS. Bound complexes were eluted by resuspension in 2× Laemmli buffer and incubation for 10 min at 90°C. Lysates and immunoprecipitates were subjected to SDS-PAGE and blotting with mouse anti-HA and mouse anti-GFP (co-immunoprecipitation of NiV-G-HA and NiV-F-GFP from HEK293T cell lysates), or with mouse anti-HA and antibodies against AP subunits (assembly of μ subunits into AP complexes in HeLa cells).

Pulse-chase metabolic labeling was performed as described [54]. Briefly, HEK293T cells grown in 150-mm dishes were transfected with constructs encoding NiV-F-AU1 or NiV-G-HA. Twenty-four hours after transfection, cells were washed and detached with PBS and spun for 3 min at 200 g and room temperature. Cells were resuspended in 12 ml of L-methionine-free DMEM/high glucose supplemented with 1 mM sodium pyruvate, 0.1% (v/v) BSA, and 25 mM HEPES pH 7.4 (methionine starvation medium) and incubated for 15 min at 37°C. Cells were pelleted and resuspended in 1.6 ml of methionine starvation medium containing 0.2 mCi/ml of [^35^S]methionine-cysteine (EasyTag Express, Perkin Elmer). Cells were subsequently labeled for 15 min at 37°C, briefly spun for 1–2 s at 14,000 g, resuspended in DMEM supplemented with 10% FBS, 2 mM L-methionine, 100 U/ml penicillin, 100 µg/ml streptomycin and 25 mM HEPES pH 7.4 (chase medium) and incubated for 5 h at 37°C with occasional stirring. Cell suspension aliquots (0.25 ml) were collected at different times of chase, diluted with cold PBS, pelleted, lysed in 0.3 ml of lysis buffer and subjected to immunoprecipitation using mouse monoclonal anti-AU1 or rabbit anti-HA as described in the preceding paragraph. Washed beads with bound complexes corresponding to the different chase times were divided into two aliquots and subjected to treatment in the presence or absence of endoglycosidase H (Endo H) (New England Biolabs) as indicated in the manufacturer's instructions. Bound proteins were eluted by addition of 2× Laemmli buffer and incubation for 10 min at 90°C followed by centrifugation for 2 min at 16,000 g and room temperature. Eluates were subjected to SDS-PAGE. Gels were fixed in methanol/acetic acid/water 40∶10∶50 by vol. for 15 min at room temperature, dried and subjected to autoradiography using Kodak BioMax MR films (Carestream Health).

### Y2H assays

Assays using the AH109 reporter yeast strain were performed as described previously [55]. Plating of transformants and positive and negative controls are described in the legend to Figure 3B.

## Supporting Information

Figure S1 **Assembly of wild-type and mutant HA-tagged μ subunits into cognate AP complexes.** (A) Wild-type (WT) and mutant AP μ subunit constructs used for transfection of neurons. All constructs contained a 10-amino acid spacer (GSGSGGSGSG) followed by a triple HA tag at their C-termini. The numbering in the scheme corresponds to the sequence of HA-tagged mouse μ1A [41). The same strategy was followed for generation of HA-tagged μ2, μ3A and μ4 WT and mutant constructs used in this study. (B) HeLa cells were transiently transfected with pCIneo-based μ constructs. Approximately 24 h after transfection, cells lysates were prepared and subjected to immunoprecipitation using rabbit (for transfections with μ1A, μ2 and μ4) or mouse (for μ3A transfections) anti-HA. Immunoprecipitated complexes were analyzed by SDS-PAGE and immunoblotting (IB) with antibodies against the large subunits of AP complexes (γ, α, β3 or ε adaptins for AP-1, AP-2, AP-3 or AP-4, respectively), as shown in the different blots. Samples of cell lysates were also subjected to SDS-PAGE and immunoblotting with mouse anti-HA antibody. The position of molecular mass markers (in kDa) is indicated at the left of blots.
(TIF)

Figure S2 **Expression of wild-type and dominant-negative mutants of μ subunits in rat hippocampal neurons.** Rat hippocampal neurons were co-transfected with NiV-F-GFP and wild-type (WT) or dominant-negative mutants of HA-tagged μ subunits (A-site mutants of μ1A, μ2, μ3A in panels A, B and C, respectively, and A- and B-site mutants of μ4 in panel D). Cells were fixed and immunostained with mouse anti-HA (to detect μ constructs) and rabbit anti-MAP2 and goat anti-ANK-G (to detect dendrites and AIS, respectively). Large images display anti-HA (green) and anti-MAP2 (red) staining (yellow indicates co-localization); insets show anti-ANK-G labeling of AIS (cyan). The AIS and axons in the large images are marked by cyan and white arrowheads, respectively. Images corresponding to NiV-F-GFP fluorescence are shown in Figure 5. Scale bar: 20 µm.
(TIF)

Figure S3 **Co-expression of μ2 D174A/W419S mutant increases surface labeling of TfR-GFP.** Rat hippocampal neurons were co-transfected on DIV4 with TfR-GFP and wild-type (WT) (left panels) or D174A/W419S HA-tagged μ2 (right panels). On DIV10 cells were fixed and incubated with anti-GFP followed by permeabilization and incubation with anti-HA antibody. Cells were subsequently immunostained with fluorescently-conjugated secondary antibodies and imaged by confocal microscopy. Grayscale images correspond to TfR-GFP staining on the cell surface (anti-GFP staining in non-permeabilized cells) (A), total TfR-GFP fluorescence (B) and HA staining (C). Images show increased surface staining of TfR-GFP in cells expressing μ2 D174A/W419S compared to cells expressing similar levels of total TfR-GFP and μ2 WT. Scale bar: 20 µm. Analysis of TfR-GFP surface staining (scored as 1, low; 2, intermediate or 3, high) in neurons expressing intermediate to high levels of WT or D174A/W419S μ2 yielded values of 1.16±0.04 (n = 88) and 2.09±0.09 (n = 89), respectively (mean±SEM of n cells) (*P*<0.01 by two-tailed Student's *t* test) (67% of neurons expressing the μ2 mutant exhibited enhanced surface levels of TfR-GFP compared to cells expressing WT μ2).
(TIF)

Figure S4 **NiV-G regulates neuronal sorting of NiV-F independently of NiV-F proteolytic activation.** (A) Scheme showing the NiV-F LVGDVR 104–109 sequence with the cleavage site (Arg 109) for cathepsin L or B [42], [29], [30]. The top scheme represents the fusion-inactive F_0_, while the bottom scheme shows the F_2_ and F_1_ forms generated upon proteolytic cleavage. Other features are as in the legend to Figure 1A. (B) Reduced cleavage of the NiV-F-Δ104–109 mutant. HEK293T cells were transiently transfected with either NiV-GFP or NiV-F-Δ104–109-GFP. Approximately 24 h after transfection, cell lysates were prepared and subjected to SDS-PAGE and immunoblotting using anti-GFP. Shown are the mobilities of the inactive NiV-F_0_ form (upper band) and the active NiV-F_1_ fragment (lower band) generated by proteolytic cleavage (the F_2_ fragment was not detected because the GFP moiety was fused to the NiV-F C-terminus). The position of molecular mass markers (in kDa) is indicated at left. (C) Somatodendritic sorting of NiV-F-Δ104–109-GFP. Rat hippocampal neurons were co-transfected with plasmids encoding NiV-F-Δ104–109-GFP and mCh-Tub, fixed and immunostained as indicated in the legend to Figure 1 B–E. The grayscale image at left represents NiV-F-Δ104–109-GFP fluorescence whereas the panel at right depicts mCh-Tub fluorescence (red) and anti-MAP2 (green) (yellow represents co-localization). The inset shows anti-ANK-G labeling (AIS shown in cyan). Cyan and red arrowheads indicate the position of the AIS and axon, respectively. The polarity index calculated for NiV-F-Δ104–109-GFP was 7.5±2.0 (Table 1). Scale bars: 20 µm. (D) Loss of NiV-F-Δ104–109-GFP polarity by NiV-G-HA. Rat hippocampal neurons were co-transfected with plasmids encoding NiV-F-Δ104–109 and NiV-G-HA. Cells were fixed and immunostained as indicated in the legend to Figure 6B. The top panels depict NiV-F-Δ104–109-GFP fluorescence (green) and anti-HA staining (red) (left and right panels, respectively). The lower left panel is a merged image of anti-MAP2 (magenta) and anti-ANK-G (cyan) staining. The lower right panel is a merged image of NiV-F-Δ104–109-GFP fluorescence (green) and anti-HA staining (red) (yellow indicates co-localization). The calculated polarity index for NiV-F-Δ104–109-GFP following co-expression of NiV-G-HA was 1.2±0.3 (Table 1). Cyan and white arrowheads map the position of the AIS and axon, respectively. Scale bar: 20 µm.
(TIF)

Figure S5 **NiV-G regulates neuronal sorting of NiV-F independently of AP-2-dependent endocytosis.** Experiments were carried out in neurons (DIV5) subjected to triple transfection for 24 or 48 h with NiV-F-GFP, NiV-G-mCh and either wild-type (WT) (left panels) or D174A/W419S HA-tagged μ2 (right panels) (longer times of triple transfection caused detrimental effects on protein expression and cell morphology). Cells were fixed and immunostained with mouse anti-HA and goat anti-ANK-G and imaged by confocal microscopy. The NiV-F-GFP and NiV-G-mCh fluorescence and their merged images are shown in A, B and C, respectively, while merged images of anti-HA and anti-ANK-G immunostaining are shown in D. The AIS and axons are marked by cyan and white arrows, respectively. Images shown correspond to cells transfected for 24 h. The D/A polarity indexes for NiV-F-GFP calculated in cells co-transfected with WT or dominant negative μ2 were 1.4±0.3 and 1.5±0.4, respectively (Table 1). In control experiments, we also measured the effects of μ2 D174A/W419S on surface levels of TfR under the same transfection conditions (DIV 5 neurons co-transfected for 24 or 48 h with TfR-GFP and WT or mutant HA-tagged μ2). Analysis of TfR-GFP surface staining (scored as 1, low; 2, intermediate or 3, high) in neurons expressing intermediate to high levels of WT or D174A/W419S μ2 yielded values of 1.17±0.04 (n = 102) and 2.61±0.07 (n = 110), respectively (mean±SEM of n cells) (*P*<0.01 by two-tailed Student's *t* test) (85% of neurons expressing the μ2 mutant exhibited enhanced surface levels of TfR-GFP compared to cells expressing WT μ2).
(TIF)

Video S1 **Live-cell imaging of axonal transport carriers in rat hippocampal neurons co-expressing NiV-F-GFP and mCh-Tubulin (top panel), NiV-G-mCh and GFP (middle panel) or NiV-F-GFP and NiV-G-mCh (bottom panel).** Images were obtained by time-lapse confocal microscopy using a spinning-disk microscope (Marianas; Intelligent Imaging). GFP and mCh constructs were exposed for 200 ms sequentially and recorded each 500 ms for 60 s.
(MOV)

Video S2 **Live-cell imaging of dendritic transport carriers in rat hippocampal neurons co-expressing NiV-F-GFP and mCh-Tubulin (top panel), NiV-G-mCh and GFP (middle panel) or NiV-F-GFP and NiV-G-mCh (bottom panel).** Images were obtained as indicated in the legend to Video S1.
(MOV)

## References

[1] Horton AC, Ehlers MD (2003) Neuronal polarity and trafficking. Neuron 40: 277–295. 14556709 10.1016/s0896-6273(03)00629-9

[2] Lasiecka ZM, Winckler B (2011) Mechanisms of polarized membrane trafficking in neurons – focusing in on endosomes. Mol Cell Neurosci 48: 278–287. 21762782 10.1016/j.mcn.2011.06.013 PMC3205304

[3] Caceres A, Ye B, Dotti CG (2012) Neuronal polarity: demarcation, growth and commitment. Curr Opin Cell Biol 24: 547–553. 22726583 10.1016/j.ceb.2012.05.011 PMC3425660

[4] McGavern DB, Kang SS (2011) Illuminating viral infections in the nervous system. Nat Rev Immunol 11: 318–329. 21508982 10.1038/nri2971 PMC5001841

[5] Koyuncu OO, Hogue IB, Enquist LW (2013) Virus infections in the nervous system. Cell Host Microbe 13: 379–393. 23601101 10.1016/j.chom.2013.03.010 PMC3647473

[6] Dotti CG, Simons K (1990) Polarized sorting of viral glycoproteins to the axon and dendrites of hippocampal neurons in culture. Cell 62: 63–72. 2163770 10.1016/0092-8674(90)90240-f

[7] Tomishima MJ, Smith GA, Enquist LW (2001) Sorting and transport of alpha herpesviruses in axons. Traffic 2: 429–436. 11422937 10.1034/j.1600-0854.2001.020701.x

[8] Ehrengruber MU, Ehler E, Billeter MA, Naim HY (2002) Measles virus spreads in rat hippocampal neurons by cell-to-cell contact and in a polarized fashion. J Virol 76: 5720–5728. 11992000 10.1128/JVI.76.11.5720-5728.2002 PMC137054

[9] Mettenleiter TC (2003) Pathogenesis of neurotropic herpesviruses: role of viral glycoproteins in neuroinvasion and transneuronal spread. Virus Res 92: 197–206. 12686430 10.1016/s0168-1702(02)00352-0

[10] Rodriguez Boulan E, Pendergast M (1980) Polarized distribution of viral envelope proteins in the plasma membrane of infected epithelial cells. Cell 20: 45–54. 6248236 10.1016/0092-8674(80)90233-0

[11] Owens RJ, Dubay JW, Hunter E, Compans RW (1991) Human immunodeficiency virus envelope protein determines the site of virus release in polarized epithelial cells. Proc Natl Acad Sci USA 88: 3987–3991. 2023946 10.1073/pnas.88.9.3987 PMC51578

[12] Naim HY, Ehler E, Billeter MA (2000) Measles virus matrix protein specifies apical virus release and glycoprotein sorting in epithelial cells. EMBO J 19: 3576–3585. 10899112 10.1093/emboj/19.14.3576 PMC313987

[13] Kolesnikova L, Ryabchikova E, Shestopalov A, Becker S (2007) Basolateral budding of Marburg virus: VP40 retargets viral glycoprotein GP to the basolateral surface. J Infect Dis 196 Suppl 2: S232–6. 17940954 10.1086/520584

[14] Wang YE, Park A, Lake M, Pentecost M, Torres B, et al (2010) Ubiquitin-regulated nuclear-cytoplasmic trafficking of the Nipah virus matrix protein is important for viral budding. PLoS Pathog 6: e1001186. 21085610 10.1371/journal.ppat.1001186 PMC2978725

[15] Lamp B, Dietzel E, Kolesnikova L, Sauerhering L, Erbar S, et al (2013) Nipah virus entry and egress from polarized epithelial cells. J Virol 87: 3143–3154. 23283941 10.1128/JVI.02696-12 PMC3592157

[16] Paton NI, Leo YS, Zaki SR, Auchus AP, Lee KE, et al (1999) Outbreak of Nipah-virus infection among abattoir workers in Singapore. Lancet 354: 1253–1256. 10520634 10.1016/S0140-6736(99)04379-2

[17] Chua KB, Bellini WJ, Rota PA, Harcourt BH, Tamin A, et al (2000) Nipah virus: a recently emergent deadly paramyxovirus. Science 288: 1432–1435. 10827955 10.1126/science.288.5470.1432

[18] Wong KT, Shieh WJ, Kumar S, Norain K, Abdullah W, et al (2002) Nipah virus infection: pathology and pathogenesis of an emerging paramyxoviral zoonosis. Am J Pathol 161: 2153–2167. 12466131 10.1016/S0002-9440(10)64493-8 PMC1850894

[19] Munster VJ, Prescott JB, Bushmaker T, Long D, Rosenke R, et al (2012) Rapid Nipah virus entry into the central nervous system of hamsters via the olfactory route. Sci Rep 2: 736. 23071900 10.1038/srep00736 PMC3471094

[20] Diederich S, Maisner A (2007) Molecular characteristics of the Nipah virus glycoproteins. Ann N Y Acad Sci 1102: 39–50. 17470910 10.1196/annals.1408.003

[21] Lee B (2007) Envelope-receptor interactions in Nipah virus pathobiology. Ann N Y Acad Sci 1102: 51–65. 17470911 10.1196/annals.1408.004 PMC7168073

[22] Lee B, Ataman ZA (2011) Modes of paramyxovirus fusion: a Henipavirus perspective. Trends Microbiol 19: 389–399. 21511478 10.1016/j.tim.2011.03.005 PMC3264399

[23] Bonaparte MI, Dimitrov AS, Bossart KN, Crameri G, Mungall BA, et al (2005) Ephrin-B2 ligand is a functional receptor for Hendra virus and Nipah virus. Proc Natl Acad Sci USA 102: 10652–10657. 15998730 10.1073/pnas.0504887102 PMC1169237

[24] Negrete OA, Levroney EL, Aguilar HC, Bertolotti-Ciarlet A, Nazarian R, et al (2005) EphrinB2 is the entry receptor for Nipah virus, an emergent deadly paramyxovirus. Nature 436: 401–405. 16007075 10.1038/nature03838

[25] Negrete OA, Wolf MC, Aguilar HC, Enterlein S, Wang W, et al (2006) Two key residues in ephrinB3 are critical for its use as an alternative receptor for Nipah virus. PLoS Pathog 2: e7. 16477309 10.1371/journal.ppat.0020007 PMC1361355

[26] Moll M, Kaufmann A, Maisner A (2004) Influence of N-glycans on processing and biological activity of the nipah virus fusion protein. J Virol 78: 7274–7278. 15194804 10.1128/JVI.78.13.7274-7278.2004 PMC421684

[27] Aguilar HC, Matreyek KA, Filone CM, Hashimi ST, Levroney EL, et al (2006) N- glycans on Nipah virus fusion protein protect against neutralization but reduce membrane fusion and viral entry. J Virol 80: 4878–4889. 16641279 10.1128/JVI.80.10.4878-4889.2006 PMC1472062

[28] Biering SB, Huang A, Vu AT, Robinson LR, Bradel-Tretheway B, et al (2012) N-Glycans on the Nipah virus attachment glycoprotein modulate fusion and viral entry as they protect against antibody neutralization. J Virol 86: 11991–12002. 22915812 10.1128/JVI.01304-12 PMC3486489

[29] Pager CT, Craft WWJ, Patch J, Dutch RE (2006) A mature and fusogenic form of the Nipah virus fusion protein requires proteolytic processing by cathepsin L. Virology 346: 251–257. 16460775 10.1016/j.virol.2006.01.007 PMC7111743

[30] Diederich S, Sauerhering L, Weis M, Altmeppen H, Schaschke N, et al (2012) Activation of the Nipah virus fusion protein in MDCK cells is mediated by cathepsin B within the endosome-recycling compartment. J Virol 86: 3736–3745. 22278224 10.1128/JVI.06628-11 PMC3302499

[31] Weise C, Erbar S, Lamp B, Vogt C, Diederich S, et al (2010) Tyrosine residues in the cytoplasmic domains affect sorting and fusion activity of the Nipah virus glycoproteins in polarized epithelial cells. J Virol 84: 7634–7641. 20484517 10.1128/JVI.02576-09 PMC2897613

[32] Erbar S, Maisner A (2010) Nipah virus infection and glycoprotein targeting in endothelial cells. Virol J 7: 305. 21054904 10.1186/1743-422X-7-305 PMC2991316

[33] Bonifacino JS, Traub LM (2003) Signals for sorting of transmembrane proteins to endosomes and lysosomes. Annu Rev Biochem 72: 395–447. 12651740 10.1146/annurev.biochem.72.121801.161800

[34] Traub LM (2009) Tickets to ride: selecting cargo for clathrin-regulated internalization. Nat Rev Mol Cell Biol 10: 583–596. 19696796 10.1038/nrm2751

[35] West AE, Neve RL, Buckley KM (1997) Identification of a somatodendritic targeting signal in the cytoplasmic domain of the transferrin receptor. J Neurosci 17: 6038–6047. 9236215 10.1523/JNEUROSCI.17-16-06038.1997 PMC6568350

[36] Farias GG, Cuitino L, Guo X, Ren X, Jarnik M, et al (2012) Signal-mediated, AP-1/clathrin-dependent sorting of transmembrane receptors to the somatodendritic domain of hippocampal neurons. Neuron 75: 810–823. 22958822 10.1016/j.neuron.2012.07.007 PMC3439821

[37] Owen DJ, Evans PR (1998) A structural explanation for the recognition of tyrosine-based endocytotic signals. Science 282: 1327–1332. 9812899 10.1126/science.282.5392.1327 PMC5600252

[38] Mardones GA, Burgos PV, Lin Y, Kloer DP, Magadan JG, et al (2013) Structural basis for the recognition of tyrosine-based sorting signals by the μ3A subunit of the AP-3 adaptor complex. J Biol Chem 288: 9563–9571. 23404500 10.1074/jbc.M113.450775 PMC3611023

[39] Burgos PV, Mardones GA, Rojas AL, daSilva LL, Prabhu Y, et al (2010) Sorting of the Alzheimer's disease amyloid precursor protein mediated by the AP-4 complex. Dev Cell 18: 425–436. 20230749 10.1016/j.devcel.2010.01.015 PMC2841041

[40] Ohno H, Tomemori T, Nakatsu F, Okazaki Y, Aguilar RC, et al (1999) Mu1B, a novel adaptor medium chain expressed in polarized epithelial cells. FEBS Lett 449: 215–220. 10338135 10.1016/s0014-5793(99)00432-9

[41] Guo X, Mattera R, Ren X, Chen Y, Retamal C, et al (2013) The adaptor protein-1 μ1B subunit expands the repertoire of basolateral sorting signal recognition in epithelial cells. Dev Cell 27: 353–366. 24229647 10.1016/j.devcel.2013.10.006 PMC3992434

[42] Moll M, Diederich S, Klenk HD, Czub M, Maisner A (2004) Ubiquitous activation of the Nipah virus fusion protein does not require a basic amino acid at the cleavage site. J Virol 78: 9705–9712. 15331703 10.1128/JVI.78.18.9705-9712.2004 PMC514977

[43] Whitman SD, Smith EC, Dutch RE (2009) Differential rates of protein folding and cellular trafficking for the Hendra virus F and G proteins: implications for F-G complex formation. J Virol 83: 8998–9001. 19553334 10.1128/JVI.00414-09 PMC2738157

[44] Jareb M, Banker G (1998) The polarized sorting of membrane proteins expressed in cultured hippocampal neurons using viral vectors. Neuron 20: 855–867. 9620691 10.1016/s0896-6273(00)80468-7

[45] Mitsui S, Saito M, Hayashi K, Mori K, Yoshihara Y (2005) A novel phenylalanine-based targeting signal directs telencephalin to neuronal dendrites. J Neurosci 25: 1122–1131. 15689548 10.1523/JNEUROSCI.3853-04.2005 PMC6725959

[46] Silverman MA, Peck R, Glover G, He C, Carlin C, et al (2005) Motifs that mediate dendritic targeting in hippocampal neurons: a comparison with basolateral targeting signals. Mol Cell Neurosci 29: 173–180. 15911342 10.1016/j.mcn.2005.02.008

[47] Gravotta D, Carvajal-Gonzalez JM, Mattera R, Deborde S, Banfelder JR, et al (2012) The clathrin adaptor AP-1A mediates basolateral polarity. Dev Cell 22: 811–823. 22516199 10.1016/j.devcel.2012.02.004 PMC3690600

[48] Kramer T, Greco TM, Taylor MP, Ambrosini AE, Cristea IM, et al (2012) Kinesin-3 mediates axonal sorting and directional transport of alphaherpesvirus particles in neurons. Cell Host Microbe 12: 806–814. 23245325 10.1016/j.chom.2012.10.013 PMC3527838

[49] Grunwald IC, Korte M, Adelmann G, Plueck A, Kullander K, et al (2004) Hippocampal plasticity requires postsynaptic ephrinBs. Nat Neurosci 7: 33–40. 14699416 10.1038/nn1164

[50] Ohno H, Fournier MC, Poy G, Bonifacino JS (1996) Structural determinants of interaction of tyrosine-based sorting signals with the adaptor medium chains. J Biol Chem 271: 29009–29015. 8910552 10.1074/jbc.271.46.29009

[51] Dell'Angelica EC, Ooi CE, Bonifacino JS (1997) Beta3A-adaptin, a subunit of the adaptor-like complex AP-3. J Biol Chem 272: 15078–15084. 9182526 10.1074/jbc.272.24.15078

[52] Caceres A, Banker G, Steward O, Binder L, Payne M (1984) MAP2 is localized to the dendrites of hippocampal neurons which develop in culture. Brain Res 315: 314–318. 6722593 10.1016/0165-3806(84)90167-6

[53] Sampo B, Kaech S, Kunz S, Banker G (2003) Two distinct mechanisms target membrane proteins to the axonal surface. Neuron 37: 611–624. 12597859 10.1016/s0896-6273(03)00058-8

[54] Bonifacino JS (1998) Protein Labeling and Immunoprecipitation. Curr Protoc Cell Biol 1: 7.1.1–7.1.10.

[55] Mattera R, Arighi CN, Lodge R, Zerial M, Bonifacino JS (2003) Divalent interaction of the GGAs with the Rabaptin-5-Rabex-5 complex. EMBO J 22: 78–88. 12505986 10.1093/emboj/cdg015 PMC140067

[56] Mattera R, Boehm M, Chaudhuri R, Prabhu Y, Bonifacino JS (2011) Conservation and diversification of dileucine signal recognition by adaptor protein (AP) complex variants. J Biol Chem 286: 2022–2030. 21097499 10.1074/jbc.M110.197178 PMC3023499

[57] Heldwein EE, Macia E, Wang J, Yin HL, Kirchhausen T, et al (2004) Crystal structure of the clathrin adaptor protein 1 core. Proc Natl Acad Sci USA 101: 14108–14113. 15377783 10.1073/pnas.0406102101 PMC521094

[58] DeLano, W.L. 2002. The PyMOL molecular graphics system. DeLano Scientific, Palo Alto, CA. http://www.pymol.org

