# Supplementary figures and images for: Co-assembly of Viral Envelope Glycoproteins Regulates Their Polarized Sorting in Neurons
Source: PLoS Pathog. 2014 May 15;10(5):e1004107. doi: 10.1371/journal.ppat.1004107 (PMC4022726; doi:10.1371/journal.ppat.1004107)

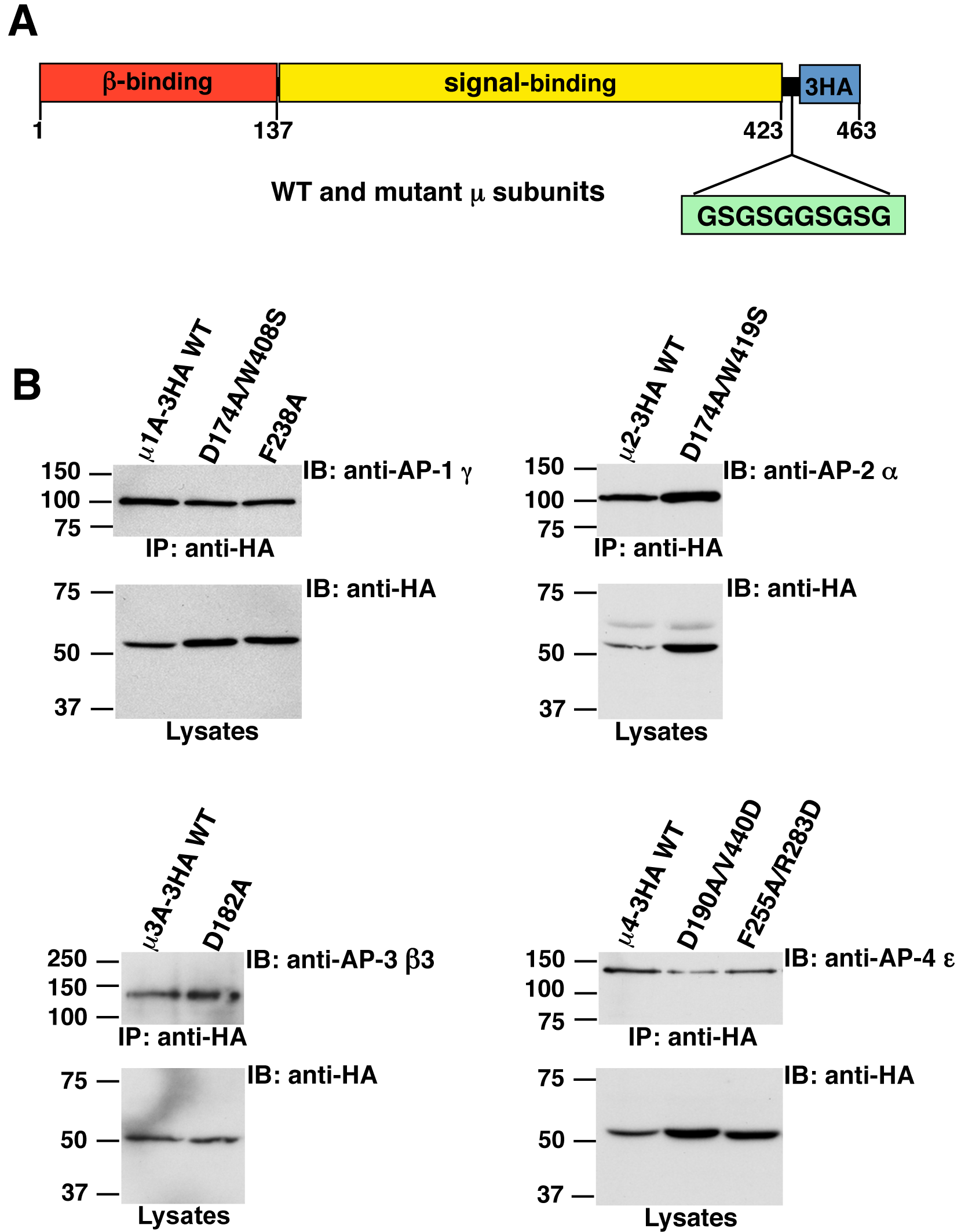

Supplement: Figure S1 — Assembly of wild-type and mutant HA-tagged μ subunits into cognate AP complexes. (A) Wild-type (WT) and mutant AP μ subunit constructs used for transfection of neurons. All constructs contained a 10-amino acid spacer (GSGSGGSGSG) followed by a triple HA tag at their C-termini. The numbering in the scheme corresponds to the sequence of HA-tagged mouse μ1A [41). The same strategy was followed for generation of HA-tagged μ2, μ3A and μ4 WT and mutant constructs used in this study. (B) HeLa cells were transiently transfected with pCIneo-based μ constructs. Approximately 24 h after transfection, cells lysates were prepared and subjected to immunoprecipitation using rabbit (for transfections with μ1A, μ2 and μ4) or mouse (for μ3A transfections) anti-HA. Immunoprecipitated complexes were analyzed by SDS-PAGE and immunoblotting (IB) with antibodies against the large subunits of AP complexes (γ, α, β3 or ε adaptins for AP-1, AP-2, AP-3 or AP-4, respectively), as shown in the different blots. Samples of cell lysates were also subjected to SDS-PAGE and immunoblotting with mouse anti-HA antibody. The position of molecular mass markers (in kDa) is indicated at the left of blots. (TIF) [file ppat.1004107.s001.tif]

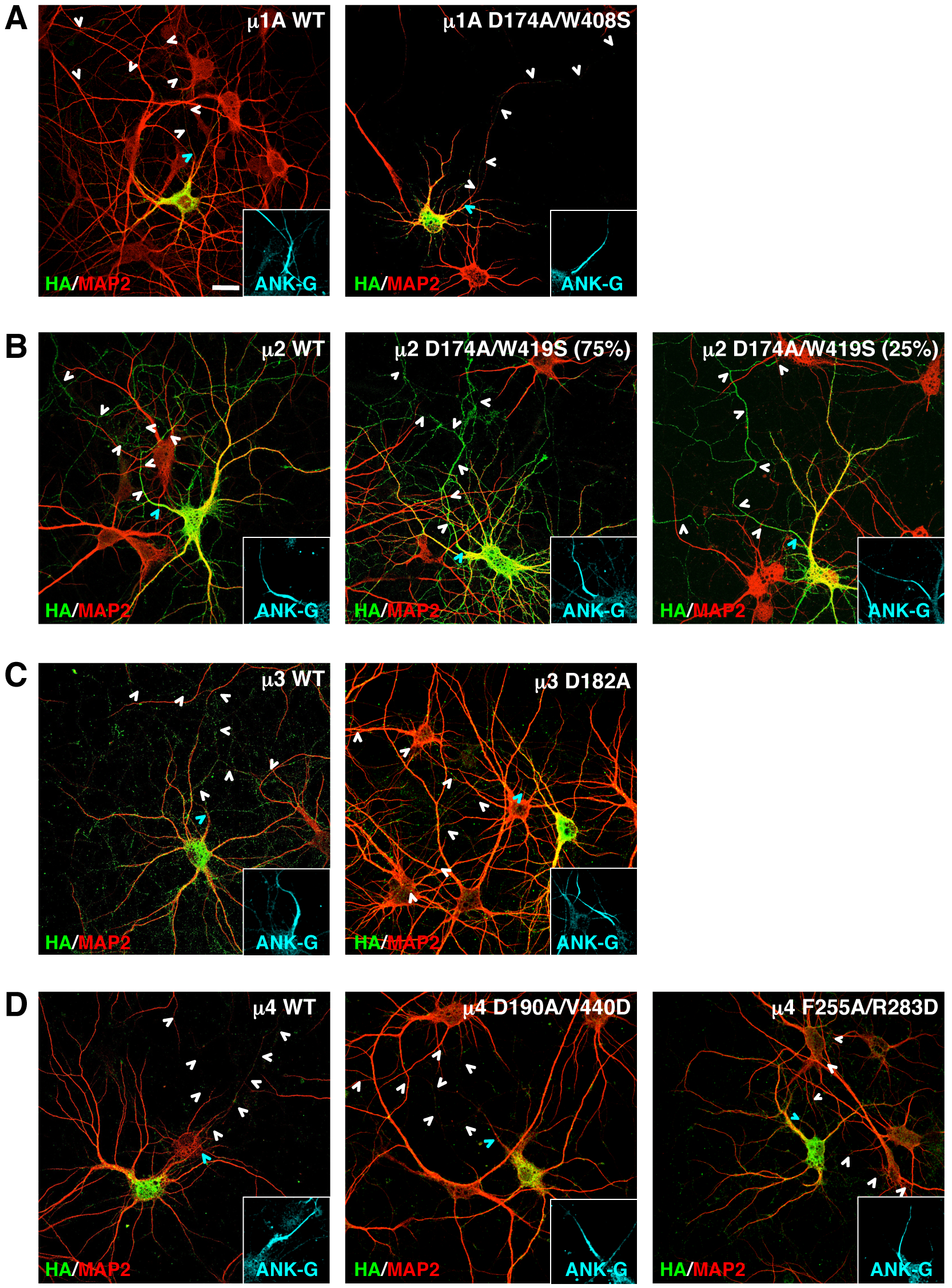

Supplement: Figure S2 — Expression of wild-type and dominant-negative mutants of μ subunits in rat hippocampal neurons. Rat hippocampal neurons were co-transfected with NiV-F-GFP and wild-type (WT) or dominant-negative mutants of HA-tagged μ subunits (A-site mutants of μ1A, μ2, μ3A in panels A, B and C, respectively, and A- and B-site mutants of μ4 in panel D). Cells were fixed and immunostained with mouse anti-HA (to detect μ constructs) and rabbit anti-MAP2 and goat anti-ANK-G (to detect dendrites and AIS, respectively). Large images display anti-HA (green) and anti-MAP2 (red) staining (yellow indicates co-localization); insets show anti-ANK-G labeling of AIS (cyan). The AIS and axons in the large images are marked by cyan and white arrowheads, respectively. Images corresponding to NiV-F-GFP fluorescence are shown in Figure 5. Scale bar: 20 µm. (TIF) [file ppat.1004107.s002.tif]

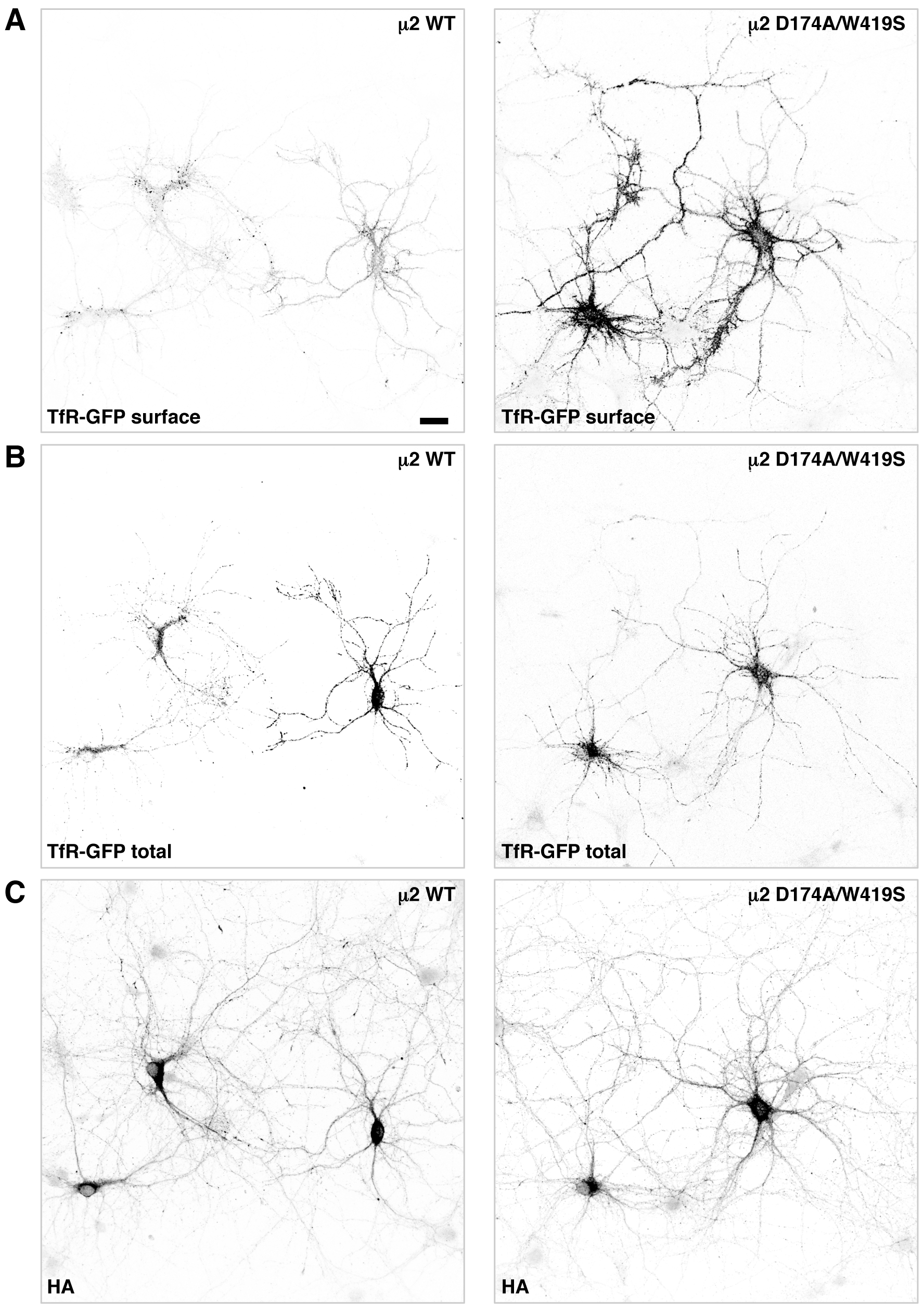

Supplement: Figure S3 — Co-expression of μ2 D174A/W419S mutant increases surface labeling of TfR-GFP. Rat hippocampal neurons were co-transfected on DIV4 with TfR-GFP and wild-type (WT) (left panels) or D174A/W419S HA-tagged μ2 (right panels). On DIV10 cells were fixed and incubated with anti-GFP followed by permeabilization and incubation with anti-HA antibody. Cells were subsequently immunostained with fluorescently-conjugated secondary antibodies and imaged by confocal microscopy. Grayscale images correspond to TfR-GFP staining on the cell surface (anti-GFP staining in non-permeabilized cells) (A), total TfR-GFP fluorescence (B) and HA staining (C). Images show increased surface staining of TfR-GFP in cells expressing μ2 D174A/W419S compared to cells expressing similar levels of total TfR-GFP and μ2 WT. Scale bar: 20 µm. Analysis of TfR-GFP surface staining (scored as 1, low; 2, intermediate or 3, high) in neurons expressing intermediate to high levels of WT or D174A/W419S μ2 yielded values of 1.16±0.04 (n = 88) and 2.09±0.09 (n = 89), respectively (mean±SEM of n cells) (P<0.01 by two-tailed Student's t test) (67% of neurons expressing the μ2 mutant exhibited enhanced surface levels of TfR-GFP compared to cells expressing WT μ2). (TIF) [file ppat.1004107.s003.tif]

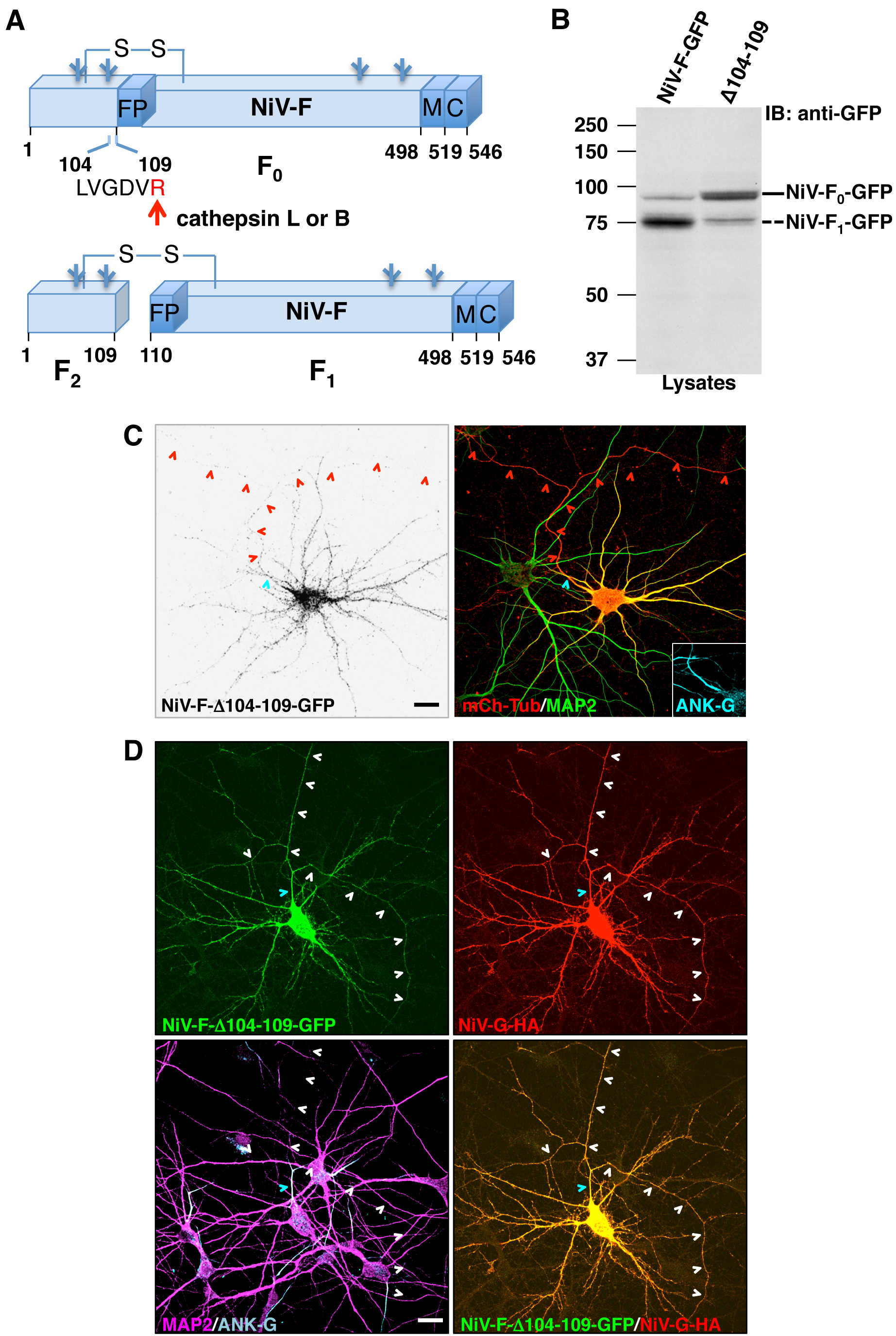

Supplement: Figure S4 — NiV-G regulates neuronal sorting of NiV-F independently of NiV-F proteolytic activation. (A) Scheme showing the NiV-F LVGDVR 104–109 sequence with the cleavage site (Arg 109) for cathepsin L or B [42], [29], [30]. The top scheme represents the fusion-inactive F0, while the bottom scheme shows the F2 and F1 forms generated upon proteolytic cleavage. Other features are as in the legend to Figure 1A. (B) Reduced cleavage of the NiV-F-Δ104–109 mutant. HEK293T cells were transiently transfected with either NiV-GFP or NiV-F-Δ104–109-GFP. Approximately 24 h after transfection, cell lysates were prepared and subjected to SDS-PAGE and immunoblotting using anti-GFP. Shown are the mobilities of the inactive NiV-F0 form (upper band) and the active NiV-F1 fragment (lower band) generated by proteolytic cleavage (the F2 fragment was not detected because the GFP moiety was fused to the NiV-F C-terminus). The position of molecular mass markers (in kDa) is indicated at left. (C) Somatodendritic sorting of NiV-F-Δ104–109-GFP. Rat hippocampal neurons were co-transfected with plasmids encoding NiV-F-Δ104–109-GFP and mCh-Tub, fixed and immunostained as indicated in the legend to Figure 1 B–E. The grayscale image at left represents NiV-F-Δ104–109-GFP fluorescence whereas the panel at right depicts mCh-Tub fluorescence (red) and anti-MAP2 (green) (yellow represents co-localization). The inset shows anti-ANK-G labeling (AIS shown in cyan). Cyan and red arrowheads indicate the position of the AIS and axon, respectively. The polarity index calculated for NiV-F-Δ104–109-GFP was 7.5±2.0 (Table 1). Scale bars: 20 µm. (D) Loss of NiV-F-Δ104–109-GFP polarity by NiV-G-HA. Rat hippocampal neurons were co-transfected with plasmids encoding NiV-F-Δ104–109 and NiV-G-HA. Cells were fixed and immunostained as indicated in the legend to Figure 6B. The top panels depict NiV-F-Δ104–109-GFP fluorescence (green) and anti-HA staining (red) (left and right panels, respectively). The lower left panel is a merged [file ppat.1004107.s004.tif]

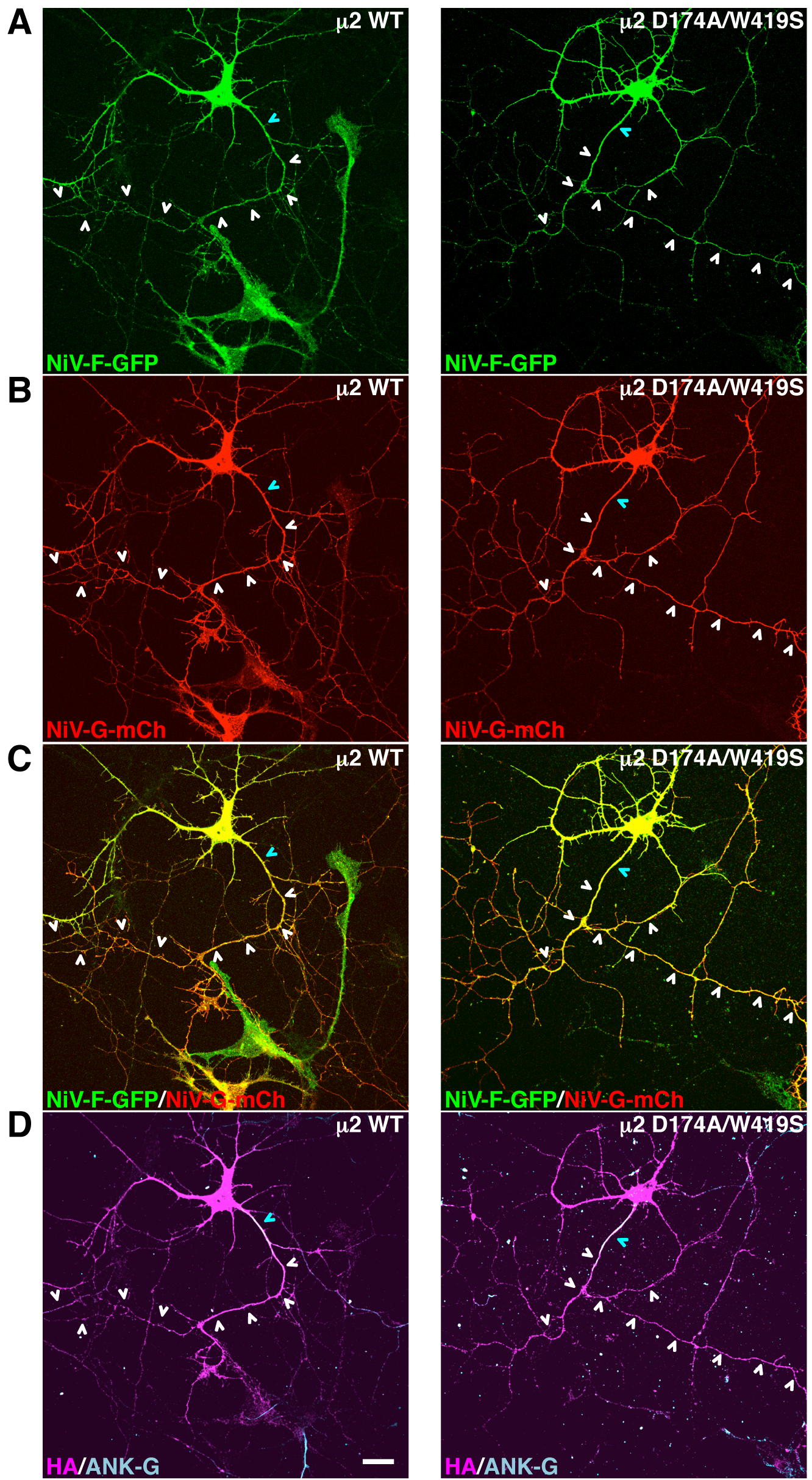

Supplement: Figure S5 — NiV-G regulates neuronal sorting of NiV-F independently of AP-2-dependent endocytosis. Experiments were carried out in neurons (DIV5) subjected to triple transfection for 24 or 48 h with NiV-F-GFP, NiV-G-mCh and either wild-type (WT) (left panels) or D174A/W419S HA-tagged μ2 (right panels) (longer times of triple transfection caused detrimental effects on protein expression and cell morphology). Cells were fixed and immunostained with mouse anti-HA and goat anti-ANK-G and imaged by confocal microscopy. The NiV-F-GFP and NiV-G-mCh fluorescence and their merged images are shown in A, B and C, respectively, while merged images of anti-HA and anti-ANK-G immunostaining are shown in D. The AIS and axons are marked by cyan and white arrows, respectively. Images shown correspond to cells transfected for 24 h. The D/A polarity indexes for NiV-F-GFP calculated in cells co-transfected with WT or dominant negative μ2 were 1.4±0.3 and 1.5±0.4, respectively (Table 1). In control experiments, we also measured the effects of μ2 D174A/W419S on surface levels of TfR under the same transfection conditions (DIV 5 neurons co-transfected for 24 or 48 h with TfR-GFP and WT or mutant HA-tagged μ2). Analysis of TfR-GFP surface staining (scored as 1, low; 2, intermediate or 3, high) in neurons expressing intermediate to high levels of WT or D174A/W419S μ2 yielded values of 1.17±0.04 (n = 102) and 2.61±0.07 (n = 110), respectively (mean±SEM of n cells) (P<0.01 by two-tailed Student's t test) (85% of neurons expressing the μ2 mutant exhibited enhanced surface levels of TfR-GFP compared to cells expressing WT μ2). (TIF) [file ppat.1004107.s005.tif]
